# Broadly neutralizing antibodies against sarbecoviruses generated by immunization of macaques with an AS03-adjuvanted COVID-19 vaccine

**DOI:** 10.1126/scitranslmed.adg7404

**Published:** 2023-05-10

**Authors:** Yupeng Feng, Meng Yuan, John M. Powers, Mengyun Hu, Jennifer E. Munt, Prabhu S. Arunachalam, Sarah R. Leist, Lorenza Bellusci, JungHyun Kim, Kaitlin R. Sprouse, Lily E. Adams, Sumana Sundaramurthy, Xueyong Zhu, Lisa M. Shirreff, Michael L. Mallory, Trevor D. Scobey, Alberto Moreno, Derek T. O’Hagan, Harry Kleanthous, Francois J. Villinger, David Veesler, Neil P. King, Mehul S. Suthar, Surender Khurana, Ralph S. Baric, Ian A. Wilson, Bali Pulendran

**Affiliations:** 1 Institute for Immunity, Transplantation and Infection, Stanford University; Stanford, CA 94305, USA.; 2 Department of Integrative Structural and Computational Biology, The Scripps Research Institute; La Jolla, CA 92037, USA.; 3 Department of Epidemiology, University of North Carolina at Chapel Hill; Chapel Hill, NC 27599, USA.; 4 Division of Viral Products, Center for Biologics Evaluation and Research, Food and Drug Administration (FDA); Silver Spring, MD 20993, USA.; 5 Sino Biological US Inc.; Wayne, PA 19087, USA.; 6 New Iberia Research Center, University of Louisiana at Lafayette; New Iberia, LA 70560, USA.; 7 Division of Infectious Diseases, Department of Medicine, Emory University School of Medicine; Atlanta, GA 30322, USA.; 8 GSK; Rockville, MD 20850, USA.; 9 Bill and Melinda Gates Foundation; Seattle, WA 98109, USA.; 10 Department of Biochemistry, University of Washington; Seattle, WA 98195, USA.; 11 Howard Hughes Medical Institute, University of Washington; Seattle, WA 98195, USA.; 12 Institute for Protein Design, University of Washington; Seattle, WA 98195, USA.; 13 Department of Pediatrics, Emory Vaccine Center, Emory National Primate Research Center; Atlanta, GA 30329, USA.; 14 Department of Pathology, Stanford University School of Medicine, Stanford University; Stanford, CA 94305, USA.; 15 Department of Microbiology and Immunology, Stanford University School of Medicine, Stanford University; Stanford, CA 94305, USA.

## Abstract

The rapid emergence of severe acute respiratory syndrome coronavirus 2 (SARS-CoV-2) variants that evade immunity elicited by vaccination has placed an imperative on the development of countermeasures that provide broad protection against SARS-CoV-2 and related sarbecoviruses. Here, we identified extremely potent monoclonal antibodies (mAbs) that neutralized multiple sarbecovirus from macaques vaccinated with AS03-adjuvanted monovalent subunit vaccines. Longitudinal analysis revealed progressive accumulation of somatic mutation in the immunoglobulin genes of antigen-specific memory B cells (MBCs) for at least one year following primary vaccination. Antibodies generated from these antigen-specific MBCs at 5 to 12 months following vaccination displayed greater potency and breadth relative to those identified at 1.4 months. Fifteen out of 338 (about 4.4%) antibodies isolated at 1.4 to 6 months after the primary vaccination showed potency against SARS-CoV-2 BA.1, despite the absence of serum BA.1 neutralization. 25F9 and 20A7 neutralized authentic clade 1 sarbecoviruses (SARS-CoV, WIV-1, SHC014, SARS-CoV-2 D614G, BA.1, Pangolin-GD) and vesicular stomatitis virus-pseudotyped clade 3 sarbecoviruses (BtKY72 and PRD-0038). 20A7 and 27A12 showed potent neutralization against all SARS-CoV-2 variants and multiple Omicron sublineages, including BA.1, BA.2, BA.3, BA.4/5, BQ.1, BQ.1.1 and XBB. Crystallography studies revealed the molecular basis of broad and potent neutralization through targeting conserved sites within the RBD. Prophylactic protection of 25F9, 20A7, and 27A12 was confirmed in mice, and administration of 25F9 in particular provided complete protection against SARS-CoV-2, BA.1, SARS-CoV, and SHC014 challenge. These data underscore the extremely potent and broad activity of these mAbs against sarbecoviruses.

## One Sentence Summary

Extremely potent broadly neutralizing antibodies against sarbecoviruses were generated by vaccination of macaques with a monovalent subunit vaccine.

## INTRODUCTION

The zoonotic spillover of coronaviruses has caused three outbreaks of severe respiratory diseases within the last 20 years (1-3). Severe acute respiratory syndrome coronavirus 2 (SARS-CoV-2), the virus that causes coronavirus disease 2019 (COVID-19), has caused an enormous global health crisis, with over 759 million confirmed cases and over 6.8 million deaths as of 7 March 2023. Although COVID-19 vaccines and therapeutic antibodies have been developed at unprecedented speed, a series of variants of concern have emerged since late 2020. The antigenically distant Omicron variant first identified in Botswana in November 2021, which has spread worldwide with multiple sublineages that evade neutralization by antibodies, has posed a serious challenge to current vaccination strategies (4-6). Recent studies have revealed that mRNA monovalent vaccine efficacy during the BA.4/5 waves was below 50% after two or three doses, with a fourth dose causing only a minimal, transient increase in Omicron-neutralizing antibodies (4-6). Furthermore, currently available therapeutic antibodies suffer from a loss of efficacy against Omicron variants (7, 8), urging the need of broadly protective vaccines (9) and monoclonal antibodies (mAbs).

We recently performed a study in macaques to benchmark clinically relevant adjuvants, (including AS03, an α-tocopherol-containing oil-in-water emulsion; AS37, a Toll-like receptor 7 (TLR7) agonist adsorbed to alum; CpG1018-alum, a TLR9 agonist formulated in alum (CpG); Essai O/W 1849101, a squalene-in-water emulsion (OW); and Alum), for their capacity to enhance the protective immunity of SARS-CoV-2 vaccines, comprising either SARS-CoV-2 RBD (RBD-NP) or a prefusion-stabilized spike protein (Hexapro-NP) on the surface of a self-assembling nanoparticle (10). We subsequently showed that a booster with RBD (beta)-NP, comprising the SARS-CoV-2 beta variant RBD, at one year later elicited robust heterotypic protection against Omicron in macaques (11). Here, we analyzed banked samples from this study to investigate the evolution of the memory B cell response at the monoclonal level over a period of 1.5 years in rhesus macaques receiving the AS03-adjuvanted subunit vaccine. We observed increased neutralization potency and breadth of mAbs isolated 6 to 12 months after the primary vaccination (the first two-dose vaccination) and 3 weeks to 6 months after the booster (the third dose vaccination at 12 months). Furthermore, we performed a serological assessment of the 15 most potent mAbs isolated 3 weeks to 6 months after the primary vaccination, performed a detailed structural analysis of a subset of these antibodies, and demonstrated their efficacy in protecting against Omicron variants of SARS-CoV-2 and other sarbecoviruses infections in mice.

## RESULTS

### AS03-adjuvanted RBD-NP/Hexapro-NP vaccination elicits progressive memory B cell maturation

We analyzed the antigen-specific, memory B cell responses from banked peripheral blood mononuclear cell (PBMC) samples from our previous study (10) in which rhesus macaques were immunized with RBD-NP adjuvanted with Alum, O/W, AS37, CpG, or AS03 (fig. S1A). We enumerated the circulating SARS-CoV-2 RBD specific IgG^+^ memory B cells (MBCs) by flow-cytometry using fluorescent-labeled probes (fig. S1B). The MBC frequency, variable (V) gene somatic hypermutation (SHM) and complementarity determining region (CDR) 3 amino-acid length were comparable among groups (fig. S1, C to E), suggesting that vaccination with these five different adjuvants exhibited a similar pattern of antigen-specific MBC responses.

**Fig. 1.**
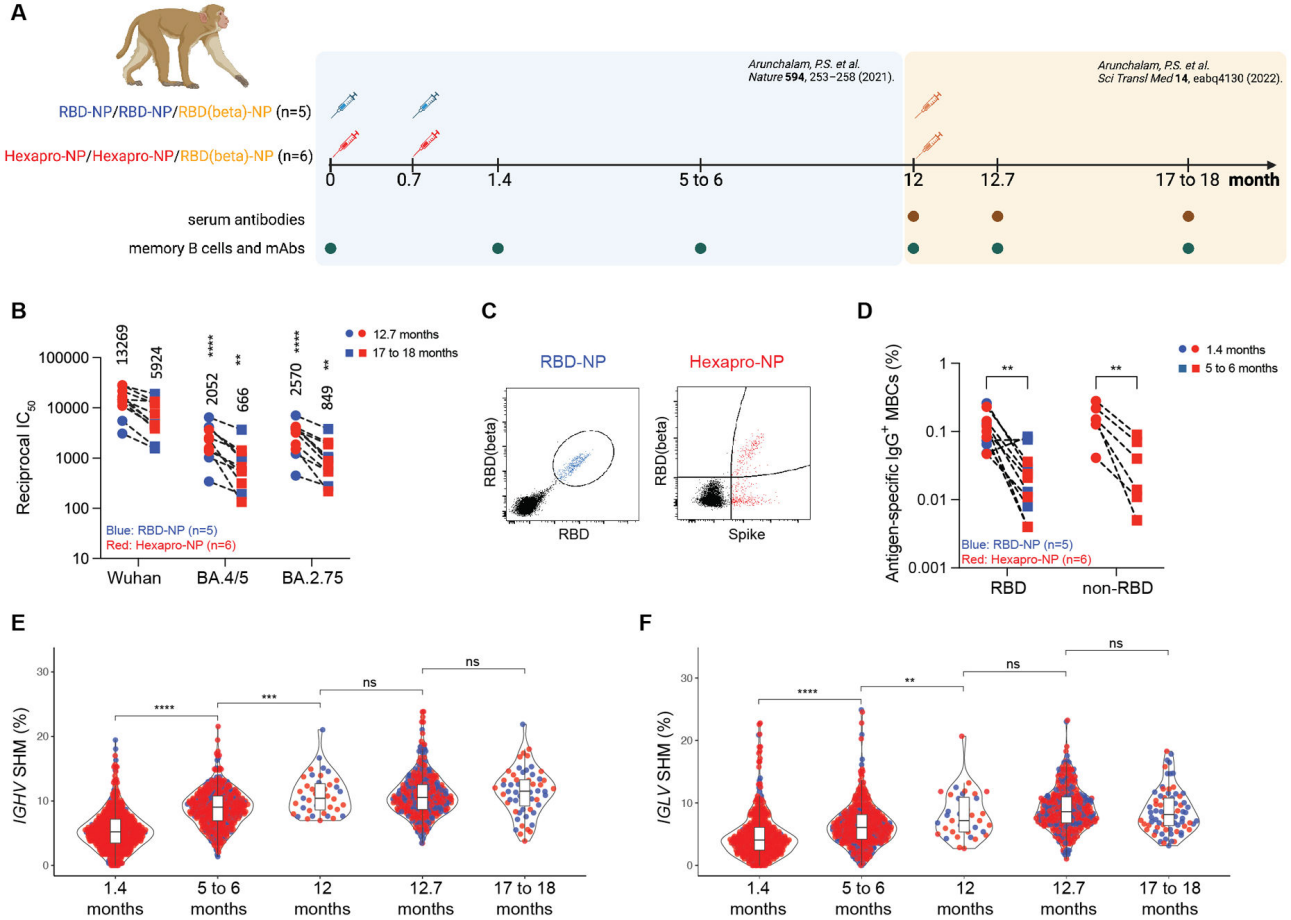
AS03-adjuvanted RBD-NP/Hexapro-NP vaccination elicits progressive memory B cell maturation. (**A**) Study overview. Rhesus macaques received AS03-adjuvanted RBD-NP or Hexapro-NP on day 0 and day 21 and received a booster with RBD (beta)-NP with AS03 at 12 months. Analysis of banked blood samples was performed as illustrated in the diagram. (**B**) Pseudovirus neutralizing antibody responses against viruses indicated on X-axis are shown. Each symbol represents an animal [RBD-NP (blue; n = 5) and Hexapro-NP (red; n = 6)], and paired samples are connected with a dashed line. The numbers within the graphs show geometric mean titers (GMTs). The statistical differences were calculated using two-way ANOVA and the statistical differences between indicated viruses and SARS-CoV-2 Wuhan strain at the same time points were labeled as (**P < 0.01 and ****P < 0.0001). (**C**) Representative flow cytometry plots show dual RBD and RBD (beta) binding B cells for RBD-NP vaccinated animals (blue), and dual spike protein and RBD (beta) binding B cells for Hexapro-NP vaccinated animals (red). Cells were pre-gated on live, CD3^−^ CD14^−^ CD16^−^ CD20^+^ IgD^−^ IgM^−^ IgG^+^ B cells. (**D**) The frequency of antigen-specific IgG^+^ MBCs relative to CD20^+^ B cells is shown for samples from the RBD-NP (blue) and Hexapro-NP (red) groups. The binding region is indicated on the X-axis. The statistical differences were calculated using two-way ANOVA (**P < 0.01). (**E and F**) Shown are the somatic hypermutation (SHM) rates of the productive *IGHV* genes (**E**) and *IGLV* genes (**F**) of B cells isolated from RBD-NP (blue) or Hexapro-NP (red) vaccinated animals at indicated time points. In (E) and (F), the boxes inside the violin plot show median, upper, and lower quartiles. The whiskers represent minimum and maximum values. Each dot represents an individual gene. The statistical differences between timepoints were calculated using one-way ANOVA (ns > 0.05, **P < 0.01, ***P < 0.001, and ****P < 0.0001).

In addition, we analyzed MBC responses using banked PBMC samples from our recent study (11) in which macaques were immunized with a two-dose primary vaccination of AS03-adjuvanted RBD-NP (n=5) or Hexapro-NP (n=6) at days 0 and 21, followed by a booster vaccination with AS03-adjuvanted RBD (beta)-NP one year later (Fig. 1A). We assessed the kinetics of the antibody response and MBCs in blood over 1.5 years in animals immunized with RBD-NP-AS03 or Hexapro-NP-AS03. Vaccination induced potent and broadly neutralizing antibodies against SARS-CoV-2 and Omicron variants after the booster (Fig. 1B and fig. S1, F and G). Omicron neutralizing antibodies were not detectable at 5 to 6 months following primary immunization, as described in our previous report (11, 12). We used flow cytometry to enumerate antigen-specific (RBD^+^ for RBD-NP group, Spike^+^ for Hexapro-NP group) MBCs in the blood (Fig. 1C and fig. S1B). The frequency of antigen-specific IgG^+^ MBCs peaked at 1.4 months and declined over the next 5 to 6 months after vaccination (Fig. 1D and fig. S2A). Immunization with Hexapro-NP elicited a high proportion of IgG^+^ MBCs targeting the non-RBD region of spike protein, although there was no difference in RBD-specific IgG^+^ MBCs between RBD-NP and Hexapro-NP (Fig. 1D).

Next, we analyzed the degree of SHM using the IMGT database (13) in the V genes of both heavy and light chain of antigen-specific MBCs, and observed a progressive increase in the frequency of SHM from 1.4 months to 5-6 months, plateauing at 12 months (Fig. 1, E and F). Booster immunization did not drive a further increase in SHM (Fig. 1, E and F). Using a more comprehensive rhesus macaque *Ig* gene database, the Karolinska Macaque database (KIMDB) (14), we similarly observed that the degree of SHM increased over time (fig. S2B). To determine the germline gene usage of anti-SARS-CoV-2 antibodies in macaques, we compared V gene nucleotide sequences of SARS-CoV-2 spike protein-specific MBCs to the *Macaca mulatta Ig* set from the IMGT database and the KIMDB database using IgBLAST. Analyses showed that *VH4-122* (IMGT) and *VH4-93* (KIMDB) were the most abundant (fig. S2, C and D). The closest germline gene of rhesus macaque *VH4-122* in humans is *VH4-59* (fig. S2E), a highly represented germline gene encoding anti-SARS-CoV-2 antibodies (15). In humans, *VH3-53* is one of the most frequently represented genes encoding antibodies generated in response to SARS-CoV-2 infection or mRNA vaccination. The structural basis of antibodies encoded by *VH3-53* has been extensively studied and showed a highly convergent binding approach to the receptor binding site (RBS) (15-17). The corresponding germline genes in rhesus macaque that are most similar to *VH3-53* are *VH3-103* (91.1%), *VH3-100* (89.9%), and *VH3S42* (89.9%), all of which have the SGGS motif in CDRH2, but no NY motif in CDRH1 (fig. S2E). Further, those human *VH3-53*-like germline genes were also abundantly represented in rhesus macaques (fig. S2C).

### Maturation of the B cell response generates antibodies with greater potency and breadth

**Fig. 2.**
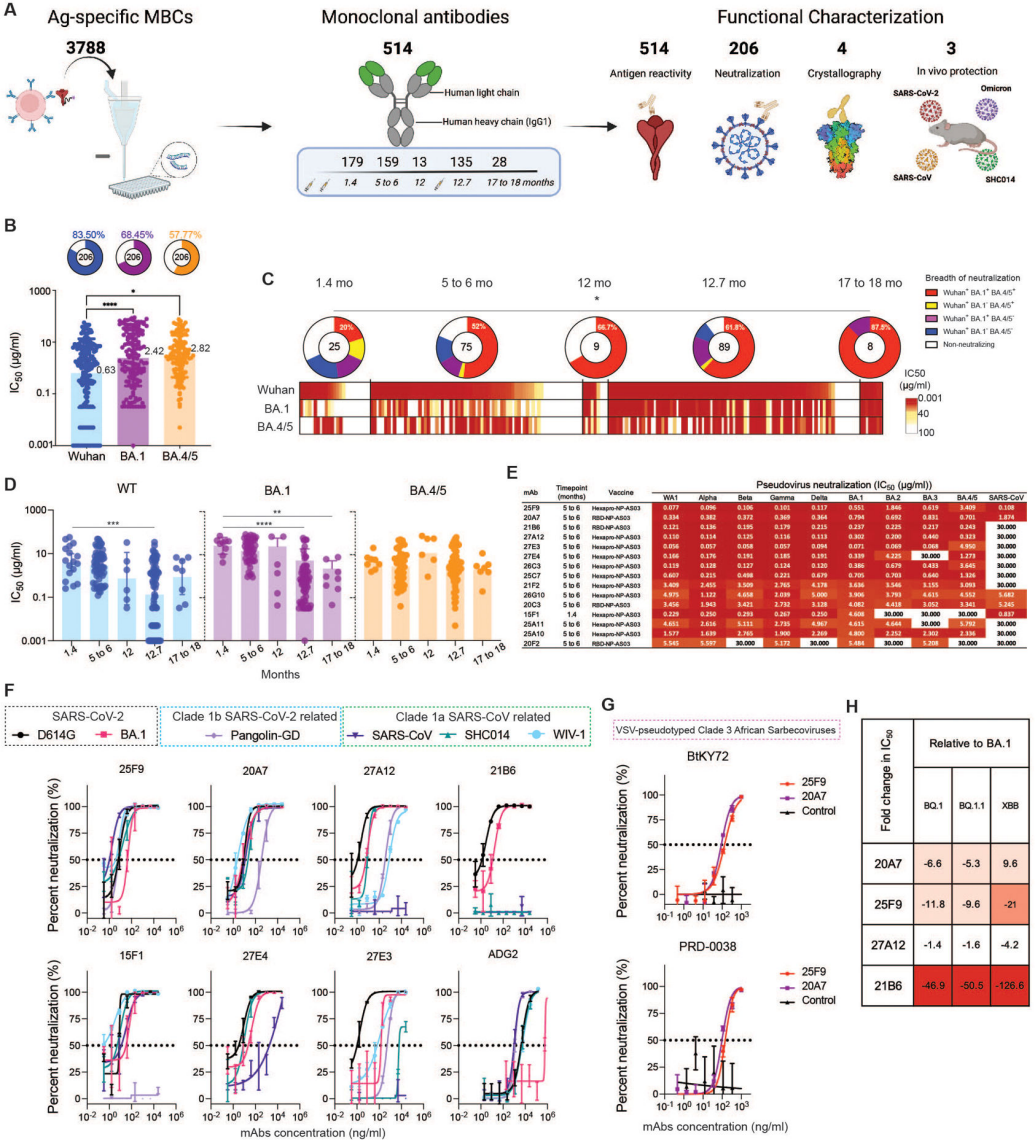
Maturation of the B cell response generates antibodies with greater potency and breadth. (**A**) Diagram depicting the strategy for antigen (Ag)-specific MBCs sorting, mAb isolation, and characterization. (**B**) Graphs showing the neutralizing activity of monoclonal antibodies measured by pseudovirus neutralization assays. Bar graphs show IC_50_s of all neutralizing antibodies against SARS-CoV-2 Wuhan (blue), BA.1 (purple), and BA.4/5 (orange) pseudoviruses. Each dot represents one antibody. Pie charts illustrate the fraction of non-neutralizing (no valid IC_50_ or IC_50_ > 100 μg/ml) antibodies (white slices); the inner circles show the number of antibodies tested. The frequency of neutralizing antibodies against SARS-CoV-2 Wuhan (blue slices), BA.1 (purple slices), or BA.4/5 (orange slices) pseudoviruses are shown on top of each pie chart, respectively. Bars and whiskers indicate geometric mean and geometric standard deviation (SD). Statistical significance was determined by the two-tailed Kruskal–Wallis test with subsequent Dunn’s multiple-comparisons test (*P < 0.05 and ****P < 0.0001). (**C**) Heatmap shows the neutralization activity of mAbs isolated at indicated time points against pseudotyped SARS-CoV-2 Wuhan, BA.1, and BA.4/5 respectively. Each unit within the heatmap represents one antibody. The color gradient indicates IC_50_ values ranging from 0.001 (red) to 100 (white). Pie charts illustrate the fraction of non-neutralizers (white slices), SARS-CoV-2 Wuhan only (blue slices), Wuhan and BA.1 double (purple slices), Wuhan and BA.4/5 double (orange slices), and Wuhan, BA.1, BA.4/5 triple (red slices) neutralizing antibodies; the inner circle shows the number of antibodies tested at indicated time points. Statistical significance between the frequencies of the five categories of antibodies isolated from five different time points was determined using a two-tailed chi-square test (*P < 0.05). (**D**) The graphs show kinetic change of the potency, reported as IC_50_ (μg/ml), of neutralizing antibodies against pseudotyped SARS-CoV-2 Wuhan (blue), BA.1 (orange) and BA.4/5 (purple), respectively. Each dot represents one antibody. Bars and whiskers indicate geometric mean and geometric SD. The statistical differences between timepoints were calculated using one-way ANOVA (**P < 0.01, ***P < 0.001 and ****P < 0.0001). (**E**) The heat map shows the IC_50_ values of 15 selected mAbs against the indicated pseudoviruses. The heatmap range from 0.01 to 30 μg/ml is represented by white to dark red. (**F**) Graphs show the neutralization of authentic SARS-CoV-2 D614, SARS-CoV-2 BA.1, Pangolin, SARS-CoV, SHC014, and WIV-1 by indicated antibodies. Symbols are means ± SD. Dashed lines indicate IC_50_ values. N=4. (**G**) 25F9 (red) and 20A7 (purple) mediated neutralization of VSV pseudoviruses containing spike proteins of Clade 3 African sarbecoviruses, BtKY72 (top) and PRD-0038 (bottom). Symbols are means ± SD. Dashed lines indicate IC_50_ values. N=3. (**H**) The fold changes in neutralization IC_50_ values of BQ.1, BQ.1.1, XBB relative to BA.1 are shown, with resistance colored from white to dark red.

To assess the evolution of the antibody repertoire encoded in antigen-specific MBCs, we sorted 3788 single SARS-CoV-2 Wuhan spike protein-specific IgG^+^ MBCs at indicated timepoints from macaques from all groups (RBD-NP plus all adjuvants and Hexapro-NP-AS03) (Fig. 1A and fig. S1A), isolated 514 mAbs, and assessed their binding and neutralization potential (Fig. 2A). Consistent with the indistinguishable SHM and CDR3 amino acid (aa) lengths (fig. S1, D and E), mAbs isolated from animals from the different adjuvanted groups displayed similar binding profiles (fig. S3A). Henceforth, all 514 mAbs were grouped per time for antibody evolution analysis.

In total, 427 out of 514 (about 83.1%) mAbs bound to the SARS-CoV-2 Wuhan spike protein measured by enzyme-linked immunosorbent assay (ELISA), and the binding capacities (area under curve, AUC) correlated weakly but significantly (R = 0.32, P < 0.0001) with the heavy chain SHM (fig. S3B). This prompted us to examine whether B cell maturation over time drove affinity maturation. As expected, binding increased over time after the primary vaccination and plateaued at 12 months (fig. S3C). We further assayed the cross-reactivities of those mAbs against BA.1 and BA.4/5 by ELISA (fig. S4A). The correlation between Omicron-binding and somatic hypermutation was absent (fig. S4, B to E). However, the proportion of WT, BA.1, BA.4/5 triple-reactive mAbs increased over time and peaked at 12 months (fig. S4F).

Next, we selected the top 206 BA.1 binding mAbs for neutralization screening against pseudotyped SARS-CoV-2 Wuhan, BA.1, and BA.4/5 strains. Of the 206 mAbs, 83.5% neutralized SARS-CoV-2 Wuhan, whereas 68.45% and 57.77% neutralized BA.1 and BA.4/5, respectively (Fig. 2B). The average potency of neutralizing antibodies against BA.1 or BA.4/5 was also significantly (*P*< 0.05 or *P*<0.0001, respectively) lower than against Wuhan strain, reflecting the fact that the vaccine contained the Wuhan strain (Fig. 2B). To determine whether antibody maturation in binding and cross-reactivity translated into enhanced neutralizing potency and breadth, we analyzed the neutralization profiles as a function of time post-immunization. There was a significant (*P*<0.05) increase in the frequency of SARS-CoV-2 Wuhan, BA.1, and BA.4/5 triple-neutralizing antibodies, and a decrease in the percentage of non-neutralizing antibodies over 1.5 years, an indication of the evolution in the neutralization potency and breadth (Fig. 2C). Further examining the consequence of this evolution, we found that the average potency of neutralization against SARS-CoV-2 Wuhan and BA.1 was significantly (*P*<0.001) improved by the booster (Fig. 2D). Collectively, our data demonstrate an evolution of mAbs in the MBC compartment towards higher neutralization potency and breadth.

### Potent broadly neutralizing antibodies (bnAbs) show pan-sarbecvorius breadth

To identify SARS-CoV-2 bnAbs, we further characterized the neutralizing antibodies isolated at 1.4 months and 5 to 6 months and identified 15 mAbs showing better or comparable neutralizing activity against BA.1 variant in a side-by-side comparison assay with a recently described BA.1 neutralizing antibody, S2H97 (18) (fig. S5A). All 15 mAbs displayed high avidities (apparent dissociation constant (K_D_) < 0.1 nM) against RBDs of different SARS-CoV-2 variants of concern and 8 mAbs showed strong avidities (K_D_: <0.1 nM to 12.4 nM) to the spike protein of SARS-CoV (Table 1). The breadth of these 15 BA.1 neutralizing antibodies was confirmed by neutralization of a panel of pseudoviruses carrying spike proteins of SARS-CoV-2 WA1, alpha, beta, gamma, delta, BA.1, BA.2, BA.3, BA.4/5, and SARS-CoV (fig. S5, B and C). Five bnAbs (25F9, 20A7, 21B6, 27A12, 27E3) stood out for their little neutralization changes across the SARS-CoV-2 variants (Fig. 2E). Remarkably, 20A7 can neutralize all SARS-CoV-2 Omicron variants and SARS-CoV with little to no reduced potency as compared to SARS-CoV-2 WA1 (Fig. 2E).

We next evaluated the neutralization of authentic SARS-CoV-2 D614G, BA.1, Pangolin-GD-CoV, SARS-CoV, SHC014, WIV-1, and MERS-CoV by 7 bnAbs (25F9, 20A7, 21B6, 27A12, 27E3, 27E4, 15F1) and a previously described ultrapotent bnAb, ADG2 (19). 25F9 neutralized all the above SARS-related viruses with half-maximal inhibitory concentration (IC_50_) values of 6 ng/ml, 42 ng/ml, 6 ng/ml, 0.85 ng/ml, 3 ng/ml, 6 ng/ml, respectively, with potencies surpassing that observed with ADG2 in a head-to-head comparison (Fig. 2F and fig. S5C). 20A7 displayed similar neutralizing breadth as compared to 25F9, albeit there was a reduction of neutralization against Pangolin-GD, whereas none neutralized MERS-CoV (fig. S5C). Furthermore, 25F9 and 20A7 displayed neutralization against vesicular stomatitis virus (VSV)-pseudotyped Clade 3 sarbecoviruses (Fig. 2G and fig. S5C), BtKY72 and PRD-0038, that were considered to have spillover potential (20). Of note, 21B6 and 27A12 neutralized BA.1 with IC_50_ values of 11 ng/ml and 5 ng/ml, respectively (Fig. 2F and fig. S5C). During the conduction of the study, new variants of concern, such as BQ.1.1 and XBB, become the dominant viruses. We assayed 25F9, 20A7, 27A12, and 21B6 for their neutralization against pseudotyped BQ.1, BQ.1.1 and XBB (Fig. 2H, and fig. S5C). 27A12 exhibited comparable neutralization potencies against BQ.1, BQ.1.1 and XBB compared to that against BA.1 (Fig. 2H). 20A7 and 25F9 showed some reduction (Fig. 2H). However, their high authentic BA.1 neutralization potencies (6 ng/ml and 42 ng/ml, respectively) (fig. S5C) make 20A7 and 25F9 competitive to recently described antibodies (7). Altogether our data revealed the cellular and molecular basis of vaccine-induced antibody evolution. More importantly, we isolated some extremely potent bnAbs (21B6, 27A12, 25F9 and 20A7) after the primary vaccination with an AS03-adjuvanted nanoparticle immunogen.

### Potent bnAbs target conserved sites within the RBD

To define the epitopes of the bnAbs and the structural basis of their neutralization breadth, we performed competitive binding experiments using antibodies (CR3022, CC12.3, CV07-270, S2X259, S2M11), whose epitopes are well characterized (15, 21-24). The top three potent Omicron neutralizers (27A12, 27E3, and 21B6) competed with the ultrapotent mAb, S2M11 (24), which recognizes epitopes overlapping with the RBS (fig. S6, A and B). Co-incubation of the top four antibodies with the greatest neutralization breadth (25F9, 20A7, 15F1, and 27E4) with CR3022 (15), a SARS-CoV neutralizing antibody, or with S2X259 (23), a pan-sarbecovirus neutralizing antibody, showed strong competition (83-96%), suggesting some similarity in their binding epitopes (fig. S6, A and B).

**Fig. 3.**
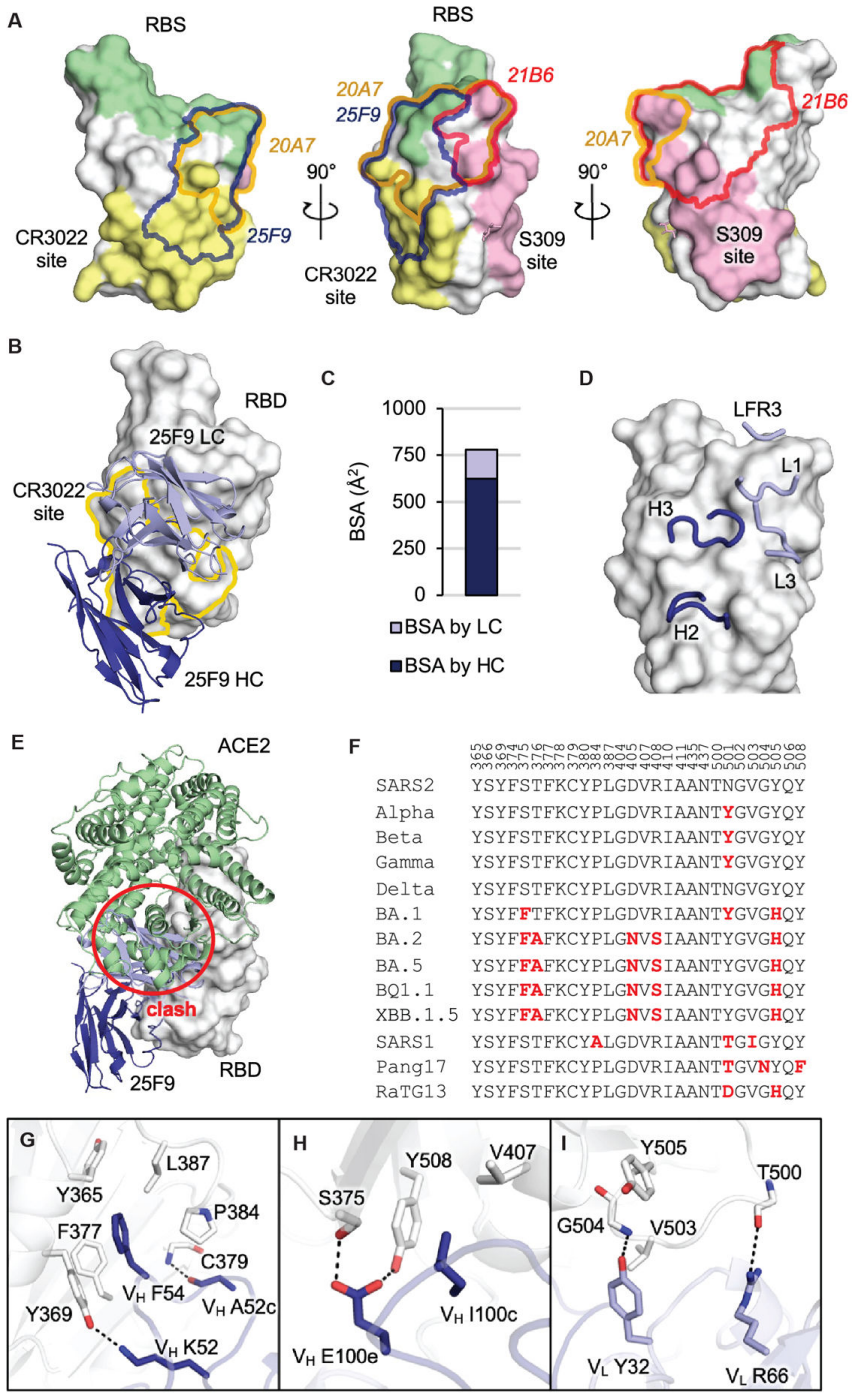
25F9 recognizes a conserved region on SARS-CoV-2 RBD. The SARS-CoV-2 RBD is shown in white and human ACE2 is in pale green throughout all the figures; the heavy and light chains of 25F9 are in blue and lavender, respectively. For clarity, only variable domains of the antibodies are shown in all figures. (**A**) Shown are the relative positions of epitopes on SARS-CoV-2 RBD (white). The RBS is shown in pale green, the CR3022 site is shown in yellow, and the S309 site is shown in pink. Epitopes of 25F9, 20A7, and 21B6 are highlighted in blue, orange, and red outlines, respectively. RBS and epitope residues are defined as buried surface area (BSA > 0 Å^2^) as calculated by Proteins, Interfaces, Structures and Assemblies (PISA, http://www.ebi.ac.uk/pdbe/prot_int/pistart.html). (**B**) Shown is the crystal structure of 25F9 in complex with SARS-CoV-2 RBD. (**C**) The surface area of SARS-CoV-2 buried by heavy and light chains of 25F9 is shown. (**D**) 25F9 interacts with RBD using CDRs H2, H3, L1, and L3, as well as LFR3. (**E**) SARS-CoV-2 RBD with 25F9 superimposed onto an RBD-ACE2 complex structure (PDB 6M0J) shows that 25F9 would clash (indicated with a red circle) with ACE2. (**F**) Shown is sequence alignment of epitope residues in a subset of SARS-like viruses. Residues that differ from wild-type SARS-CoV-2 are indicated in red. SARS2, wild-type SARS-CoV-2; SARS1, SARS-CoV-1. (**G to I**) Molecular interactions between RBD and CDR H2 (G), CDR H3 (H), and light chain (I) are shown. Hydrogen bonds and salt bridges are indicated by dashed lines.

**Fig. 4.**
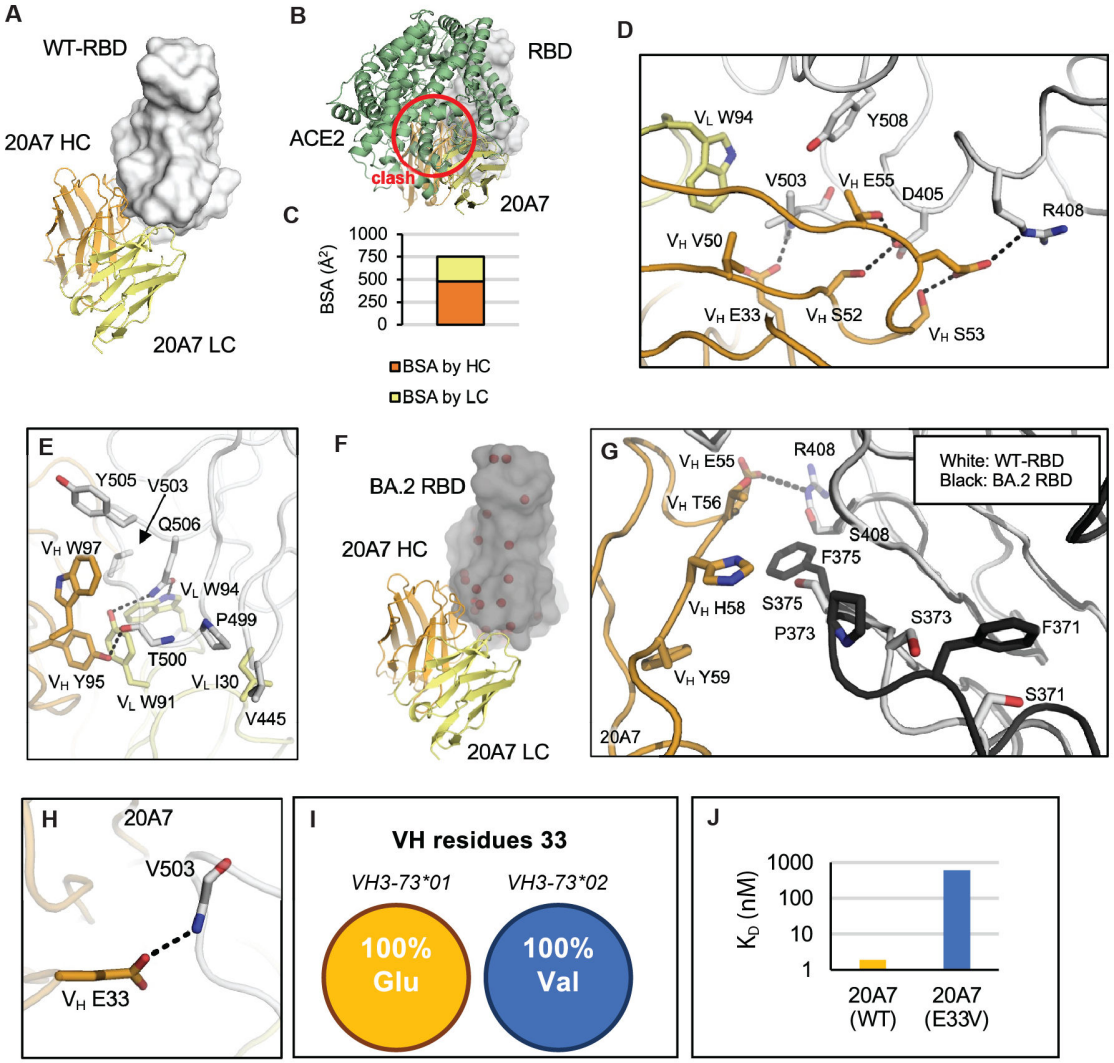
20A7 accommodates mutations of SARS-CoV-2 variants. The SARS-CoV-2 wild-type and BA.2 RBDs are shown in white and black; the heavy and light chains of 20A7 are in orange and yellow. Hydrogen bonds and salt bridges are indicated by dashed lines. (**A**) Shown is the crystal structure of 20A7 in complex with SARS-CoV-2 wild-type RBD. (**B**) SARS-CoV-2 RBD in complex with 20A7 superimposed onto an RBD-ACE2 complex structure (PDB 6M0J) shows that 20A7 would clash (red circle) with ACE2. (**C**) Shown is the surface area of SARS-CoV-2 wild-type RBD that would be buried by heavy and light chains of 20A7. (**D**) Molecular interactions between wild-type SARS-CoV-2 RBD and 21B6 CDR H2. (**E**) Molecular interactions between wild-type SARS-CoV-2 RBD and 21B6 CDR H3. (**F**) The crystal structure of 20A7 with RBD (BA.2) shows that 20A7 targets BA.2 in the same binding mode as wild-type SARS-CoV-2. Mutated residues in the Omicron BA.2 subvariant are indicated by red spheres. (**G**) Shown is a structural comparison of the interaction of 20A7 with wild-type (white) and BA.2 (black) RBDs. (**H**) 20A7 V_H_ E33 forms a hydrogen bond with RBD. (**I**) All alleles of VH3-73*01 encode Glu at position 33 whereas those of VH3-73*02 encode Val. (**J**) Biolayer interferometry (BLI) binding assay results showed that E33V reduced the binding of 20A7 to SARS-CoV-2 RBD by about 300-fold.

Next, we applied X-ray crystallography to determine the crystal structures of SARS-CoV-2 RBD in complex with three antibodies isolated in this study (table S1), 25F9 (Fig. 3), 20A7 (Fig. 4), and 21B6 (fig. S7). Relative positions of epitopes of the three antibodies as well as the RBS, the CR3022 site, and the S309 (25) site are shown in Fig. 3A. 25F9 targets one side of the RBD with some overlap with the conserved CR3022 site (Fig. 3B), where approximately 80% of the 25F9 epitope is buried by the heavy chain (Fig. 3C). Heavy chain (H) and light chain (L) CDRs H2, H3, L1, L3, and light chain framework region 3 (LFR3) interact with RBD (Fig. 3D). 25F9 binding would clash with the human angiotensin converting enzyme 2 (ACE2) (Fig. 3E), which explains its high potency. 25F9 targets a conserved region of the RBD, where 23 to 27 out of 28 epitope residues are conserved among SARS-CoV-2 variants, including BQ.1.1 and XBB.1.5, and other SARS-like viruses, such as SARS-CoV (SARS1), pang17, and RaTG13 (Fig. 3F), which further explains the high potency of 25F9 against a broad range of SARS-like viruses (Fig. 2, E and F). 25F9 V_H_ F54 inserts into a hydrophobic pocket in the RBD and stacks with aromatic residues RBD-Y365, F377, Y369, P384, and aliphatic residue L387 (Fig. 3G). 25F9-V_H_ K52 side chain and V_H_ A52c backbone carbonyl hydrogen bond with RBD-Y369 side-chain hydroxyl and C379 backbone amide, respectively (Fig. 3G). The CDR H3 and light chain of 25F9 also form polar and hydrophobic interactions with the RBD (Fig. 3, H and I).

21B6 neutralizes a broad range of SARS-CoV-2 variants, including BA.2 and BA.5, with high potency, but does not neutralize other SARS-like viruses SARS-CoV and SHC014 (Fig. 2, E and F). In contrast, 21B6 binds the opposite side (above the S309 epitope) of SARS-CoV-2 RBD (fig. S7A). Like 25F9, 21B6 targets RBD with approximately 80% of its epitope area buried by the heavy chain (fig. S7B), and CDRs H1, H2, H3, L2, and heavy chain framework region 1 (HFR1) interact with the RBD (fig. S7C). 21B6 would also clash with ACE2 (fig. S7D). The 21B6 epitope residues are conserved from wild-type SARS-CoV-2 through Omicron subvariants BA.2 and BA.5, but vary in other SARS-like viruses (fig. S7E). RBD-Y351 hydrogen bonds with 21B6-V_H_ S30 and D31 and makes hydrophobic interactions with V_H_ T28 and Y32 (fig. S7F). CDR H2, especially V_H_ V52b and L52c, extensively interact with a hydrophobic patch on RBD formed by L452, Y351, L492, F490, and T470 (fig. S7G). CDR H3 and the light chain of 21B6 also form extensive interactions with the RBD (fig. S7, H and I). The R346T mutation in the most recent circulating Omicron subvariants BQ.1.1 and XBB.1.5 would cause a loss of salt bridge with 21B6-V_H_ D101, which may contribute to some loss of neutralization (Fig. 2G).

20A7 neutralized all SARS-CoV-2 variants tested as well as SARS-CoV (Fig. 2, E and F). The crystal structure of 20A7 with wild-type SARS-CoV-2 RBD (Fig. 4A) shows its binding to RBD would clash with ACE2 (Fig. 4B). Both heavy and light chains interact with the RBD, where the relative RBD surface area buried by heavy and light chains is approximately 2/3 and 1/3, respectively (Fig. 4C). CDRs H2 (Fig. 4D) and H3 (Fig. 4E) form extensive interactions with the RBD. 20A7 neutralizes Omicron subvariants with little reduction in activity (Fig. 2, E and F). We also determined a crystal structure of 20A7 in complex with Omicron BA.2 RBD (Fig. 4F) that exhibited the same binding mode as with wild-type SARS-CoV-2 RBD (Fig. 4A). Comparison of the interactions of 20A7 with wild-type and BA.2 variant (Fig. 4G) showed that the salt bridge formed by 20A7 V_H_ E55 and RBD-R408 was eliminated by the R408S mutation in BA.2, which may contribute to some loss of binding affinity. In addition, mutations at S371, S373, and S375 in the Omicron subvariants induce a localized conformational change in the main chain of a loop containing these residues in the RBD (26).

Some of the authors of this study previously determined two antibody structures encoded by a highly enriched germline gene *VH3-73* in macaques (27). In the present study, we show that another *VH3-73*-encoded antibody, 20A7, adopts the same binding mode (fig. S8, A to C). Importantly, V_H_ E33 in all three antibodies forms hydrogen bonds with the backbone amide of RBD-V503. V_H_ E33 is only encoded by the alleles clustered as VH3-73*01. In contrast, VH3-73*02 alleles encode a valine instead of glutamic acid. This single mutation in 20A7, V_H_ E33V, markedly reduced the binding affinity by about 300-fold, presumably due to the loss of this hydrogen bond (Fig. 4, H to J, and fig. S8), suggesting that it may be difficult to generate such bnAbs in macaques that do not have an VH3-73*01 allele. We previously made a similar observation that human anti-SARS-CoV-2 *VH2-5/VL2-14* antibodies are also allele-specific (28). These observations highlight the importance of considering allele polymorphism in vaccine design.

We previously discovered a broad-and-potent neutralization epitope on the RBDs of SARS-related coronaviruses (29). The epitope spans from a corner of RBS-D to the CR3022 site (fig. S9). Here we show that the broad-and-potent neutralizing antibodies 25F9 and 20A7 also target this RBS-D/CR3022 site and neutralize all tested SARS-CoV-2 variants as well as other SARS-like viruses. Furthermore, a neutralizing antibody SA55 in a clinical trial potently neutralizes all known SARS-CoV-2 variants including BQ.1.1 and XBB and also targets the RBS-D/CR3022 site (30) (fig. S9). The features of this site that elicit recurring broad and potent neutralizing antibodies provide a promising target for universal COVID-19 vaccines.

### Potent bnAbs show broad sarbecovirus protection in mice

**Fig. 5.**
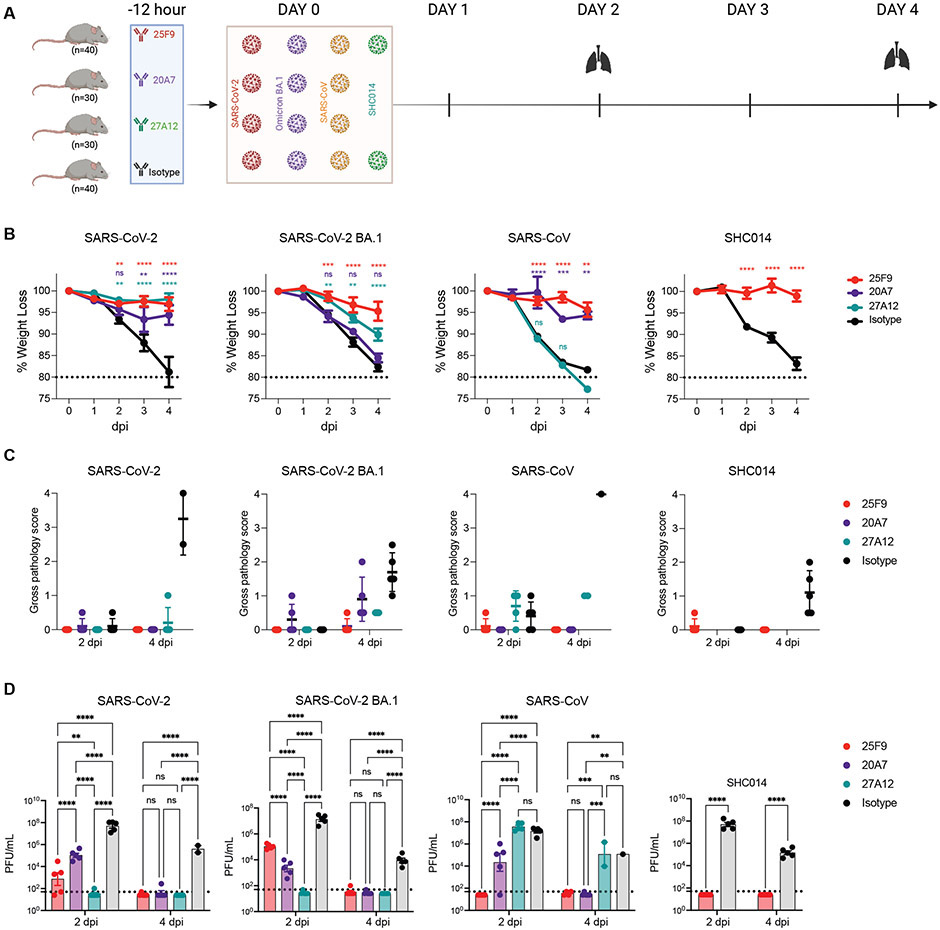
25F9, 20A7, and 27A12 protect aged mice from SARS-CoV-2 and other sarbecovirus-induced pathology. (**A**) Shown is a diagram depicting the challenge study in mice. 25F9, 20A7, 27A12, or a DENV2(2D22) control antibody were administered intraperitoneally at 200 μg per animal into 14 groups of aged mice (10 animals per group). Animals were challenged intranasally 12 hours after antibody infusion with one of the indicated sarbecoviruses (mouse-adapted SARS-CoV-2, 1 × 10^3^ PFU; mouse-adapted SARS-CoV-2-BA.1, 1 × 10^5^ PFU; mouse-adapted SARS-CoV, 1 × 10^4^ PFU; or SHC014 MA15, 1 × 10^5^ PFU). Lungs were collected on day 2 or 4 after infection. As a control, groups of mice were exposed only to phosphate-buffered saline (PBS) in the absence of virus. (**B**) Shown is the body weight change of mice after challenge with moused-adapted SARS-CoV-2, SARS-CoV-2 BA.1, SARS-CoV, and SHC014, respectively. Data are presented as mean ± SEM from 10 animals per group from days 0 to 2, or 5 animals per group from days 3 to 4. Data were analyzed using a mixed-effects model with post hoc Dunnett’s multiple tests in comparison with the control group; significance is indicated as **p < 0.01, ***p < 0.001, and ****p < 0.0001 or ns when not significant. The dotted horizontal line at 80% designates a weight loss amount at which softened mouse food is added to the cages. (**C**) Lung gross pathology was scored at the collection on day 2 and 4 post-infection in mice prophylactically treated with indicated bnAbs or the isotype control mAb (n = 5 per group). Individual mice are represented by the dot plots. Data are presented as mean values ± SEM. (**D**) Lung virus titers (PFU per lung) were determined by plaque assay of lung tissues collected at days 2 or 4 after infection (n = 5 individuals per time point for each group). Data are shown as scatter dot plots with bar heights representing the mean and whiskers representing SEM. Data were analyzed with a mixed-effects model with post hoc Dunnett’s multiple tests in comparison with the control group; significance is indicated as **p < 0.01, ***p < 0.001, and ****p < 0.0001 or ns when not significant. The dotted horizontal line indicates the limit of detection (50 PFU) for the plaque assay. For samples with values below this, data is plotted at half the limit of detection.

To evaluate the protection conferred by the best bnAbs (25F9 and 20A7) and the best BA.1-neutralizing mAb (27A12), we conducted prophylactic challenge studies in 12 months old female BALB/c mice with mouse-adapted (MA) sarbecoviruses, including SARS-CoV-2 (MA10), SARS-CoV-2 BA.1, and SARS-CoV (MA15) (31-33). We conducted an additional SHC014 challenge study (34) for 25F9 because of its extensive neutralization breadth and potency (Fig. 5A). mAbs were administered by intraperitoneal injection at 200 μg per mouse 12 hours before intranasal administration of viruses with 10^3^ plaque-forming units (PFU) for SARS-CoV-2 (MA10), 10^4^ PFU for SARS-CoV (MA15), and 10^5^ PFU for SARS-CoV-2 BA.1 and SHC014 MA15. Mice were monitored for daily weight changes and lung tissues were collected two- or four-days post-infection (dpi) for gross pathology assessment and virus quantification analysis. Mice treated with isotype control antibody exhibited substantial and progressive weight loss due to the infection with all viruses. 25F9 completely prevented weight loss from SARS-CoV-2 (MA10), SARS-CoV (MA15), and SHC014 MA15 infection (Fig. 5B). Although 20A7 and 27A12 showed much higher BA.1 neutralization than that of 25F9 (IC_50_: 6 ng/ml, 5 ng/ml, and 42 ng/ml, respectively) in vitro (fig. S5C), 25F9 showed the best protection from weight loss after BA.1 infection (Fig. 5B). No signs of lung discoloration (gross pathology) were observed at either 2 or 4 dpi in mice treated with 25F9 (Fig. 5C). Furthermore, we assessed the viral load in the lungs. 25F9 completely abrogated viral replication in all mice at 4 dpi (Fig. 5D). Consistent with in vitro neutralization, 20A7 and 27A12 prophylactic treatment leads to better BA.1 clearance in vivo than 25F9 at 2 dpi (Fig. 5D), suggesting 25F9 potentially utilized multi-function for disease protection. We concluded that all three mAbs effectively protected against SARS-CoV-2 (MA10) infection in mice. 25F9 protected against the SARS-CoV-2 BA.1 and other sarbecoviruses equally effectively as it protected against the SARS-CoV-2 (MA10), highlighting its potential as a broad and potent sarbecovirus prophylactic antibody.

## DISCUSSION

Memory B cells mature over time after SARS-CoV-2 infection or mRNA vaccination (34-38). We recently showed that an AS03, a squalene oil-in-water emulsion adjuvant developed by GlaxoSmithKline, adjuvanted nanoparticle vaccine conferred durable and heterotypic protection against Omicron challenge with 100% and about 65% protection at 6 weeks and 6 months post the booster, respectively (11). The rapid elicitation of bnAbs in serum following the booster suggested the evolution of a broad and potent antibody repertoire encoded in the memory B cell compartment. Consistent with this notion, we found in this study that somatic hypermutations and the potency and breadth of antibodies encoded by B cell receptors in MBCs evolved after the primary vaccination. Those matured MBCs with greater potency and breadth can rapidly differentiate into antibody-secreting cells in response to a booster immunization or infection. Although it is well-known that adjuvants can modulate and enhance the magnitude, breadth, and durability of the vaccine-induced serum antibody response, few studies have investigated their effects on the monoclonal level (28, 40-46). In this study, we found the primary vaccination of the AS03-adjuvanted nanoparticle-based subunit vaccine elicited a progressive antibody evolution towards greater potency and breadth over a period of one year, presumably driven by antigen-antibody complexes on follicular dendritic cells.

Due to the scarcity of effective mAbs against the Omicron variants (7, 8, 47-49) and the potential for zoonotic coronaviruses such as SARS-CoV and MERS-CoV, as well as bat coronaviruses like WIV-1, RaTG13, and SHC014 (2, 3, 33, 50-54), to spill-over into humans, considerable effort is focused on identifying broadly neutralizing antibodies able to cross-neutralize various SARS-CoV-2 variants and other SARS-related viruses. However, there is always a compromise between potency and breadth (17, 19, 47, 55-57). Here we identified 7 bnAbs showing potent neutralization against authentic SARS-CoV-2 WA1 strain with IC_50_ values below 10 ng/ml. All 7 mAbs neutralized previous SARS-CoV-2 variants of concern without any reduction in potency. 25F9, 20A7, 21B6, and 27A12 neutralized authentic SARS-CoV-2 BA.1 with IC_50_ values of 42 ng/ml, 6 ng/ml, 11 ng/ml, 5 ng/ml, respectively. Notably, 27A12 showed little if any reduction in their neutralization against SARS-CoV-2 BA.2, BA.3, BA.4/5, BQ.1, BQ.1.1 and XBB relative to BA.1. 25F9 and 20A7 neutralized authentic SARS-CoV and several other bat coronaviruses with comparable potency as compared to that against SARS-CoV-2, albeit 20A7 showed some reduction of neutralization against a Pangolin strain. Furthermore, we determined crystal structures of mAbs (25F9, 20A7, and 21B6) and their mode of binding to the RBD of SARS-CoV-2, as well as one structure of 21B6 complexed with the RBD of SARS-CoV-2 BA.2, at resolutions of 3.05, 2.58, 1.75 and 2.30 Å, respectively. Interestingly, despite targeting the same overall region in the RBD, namely ‘RBS-D/CR3022’ (28) (fig. S10), antibodies such as ADG20, DH1047, and S2X259) (29, 47) lost neutralization potency against Omicron and its subvariants. In contrast, other antibodies such as 20A7, identified in this study, and SA55 are largely resistant to mutations observed in the Omicron subvariants. Multiple factors can affect resistance or loss of neutralization potency of antibodies, including but not limited to decreases in binding affinity, angles of approach to an epitope, and dependence on particular residues in the epitope that might affect the binding of one antibody and not another. Nevertheless, as we and others have shown, this region in the RBD has the potential to elicit broad and potent antibodies. Thus, molecular understanding of how different antibodies are able to target this region can provide valuable insights for epitope-focused, next-generation vaccine design.

Finally, 25F9, 20A7, and 27A12 were evaluated for their prophylactic protection efficacy against four different SARS-related viruses, including mouse-adapted SARS-CoV-2 MA10, SARS-CoV-2 BA.1, SARS-CoV MA15 and SHC014 MA15 in aged mice. This mouse model is well recognized for its efficient recapitulation of disease pathogenesis (58). Our results demonstrated that prophylactic treatment with a single dose of bnAbs not only led to clinical improvement, as shown by the absence of weight loss, but also to markedly reduced lung pathology, virus load, and inflammatory infiltration. Rhesus macaque derived antibodies have some potential issues of immunogenicity in humans. However, animal-derived antibodies have been widely used in clinical trials, such as mouse-derived humanized antibodies, Trastuzumab, Bevacizumab, and Natalizumab (59). Furthermore, non-human primates are the closest living relatives of human beings, which could have better performance than mouse-derived mAbs in clinical use.

Our study has some limitations. A direct longitudinal comparison between a non-adjuvanted and adjuvanted group of vaccinees, could not be made because of the unavailability of blood samples. Whether the same degree of antibody maturation and bnAbs will be observed without an adjuvant or with other adjuvants, or in humans, needs further investigation. Also, it will be vitally important to identify antibody escape mutants and evaluate the emergence in vivo of pathogenic variants in the presence of antibody. The focus of the current study is the isolation and extensive characterization of MBC-derived monoclonal antibodies that demonstrate potent and broad neutralization capacity despite a lack of such neutralization activity in serum, as shown in the summary of our study (fig. S11). In conclusion, we identified 25F9 and 20A7 as two highly potent and broadly neutralizing antibodies, making them promising prophylactic candidates against sarbecovirus infection.

## Materials and Methods

### Study Design

The study used banked blood samples from our previous study (10, 11), in which two groups of male rhesus macaques (Macaca Mulatta) were involved. The first group of the five animals received two doses of RBD-Wu and the second group comprising six animals received two doses of HexaPro (HexaPro Spike protein from the ancestral strain displayed on I53-50 nanoparticles). Both immunogens were administered with the AS03 adjuvant on days 0 and 21 using a prime-boost regimen. All 11 animals from both groups were boosted with an I53-50 nanoparticle immunogen displaying RBD from the beta variant about a year after the first immunization series. The number of animals in each group was determined to identify large differences between groups based on our previous experience. We did not do a power calculation to determine the sample size. The animals were randomly distributed between groups, considering body weight and age as the critical variables. The investigators were not blinded to allocation during experiments and outcome assessment.

### Statistical analysis

Individual-level data for all the figures are presented in Data file S1. The difference between any two groups at a time point was measured using a two-tailed nonparametric Mann-Whitney unpaired rank-sum test or the two-tailed Kruskal–Wallis test with subsequent Dunn’s multiple-comparisons test. The difference between groups at different time points was measured using two-way ANOVA. The difference between multiple time points was measured using one-way ANOVA. The difference between different categories was measured by the two-tailed chi-square test. All correlations were Spearman’s correlations based on ranks. All statistical analyses were performed using GraphPad Prism v.9.0.0 or R version 3.6.1. All the figures were made in GraphPad Prism or R and organized in Adobe Illustrator.

## Supplementary Material

Supplemental Material

adg7404_Data_file_S1 3

## Figures and Tables

**Table 1.** Shown are binding avidities of indicated mAbs against RBDs of SARS-CoV-2 wild-type (WT), Omicron BA.1, Alpha, Beta, Delta variants and spike proteins of SARS-CoV.

mAb	Binding avidities measured by Biolayer interferometry (Apparent K_D_, nM)					
	RBD (WT)	RBD (BA.1)	RBD (Alpha)	RBD (Beta)	RBD (Delta)	Spike (SARS-CoV)
25F9	<0.1	<0.1	<0.1	<0.1	<0.1	<0.1
20A7	<0.1	<0.1	<0.1	<0.1	<0.1	9.390
21B6	<0.1	<0.1	<0.1	<0.1	4.100	n.a.
27A12	<0.1	0.300	<0.1	<0.1	<0.1	n.a.
27E3	<0.1	14.400	<0.1	18.900	<0.1	n.a.
27E4	<0.1	<0.1	<0.1	<0.1	<0.1	12.400
26C3	<0.1	11.400	<0.1	2.000	<0.1	n.a.
25C7	<0.1	<0.1	<0.1	<0.1	<0.1	1.620
21F2	<0.1	<0.1	<0.1	<0.1	<0.1	n.a.
26G10	<0.1	<0.1	<0.1	<0.1	<0.1	0.570
20C3	<0.1	<0.1	<0.1	<0.1	<0.1	1.000
15F1	1.000	<0.1	<0.1	<0.1	<0.1	6.380
25A11	<0.1	<0.1	<0.1	<0.1	<0.1	n.a.
25A10	<0.1	<0.1	<0.1	<0.1	<0.1	n.a.
20F2	0.100	<0.1	<0.1	<0.1	<0.1	3.350
CR3022	14.100	17.400	12.000	12.700	8.300	n.a.
CC12.3	5.800	n.b.	n.a.	n.a.	n.a.	n.a.

n.b. = no binding

n.a. = data not available
