# Supplementary material for: Broadly neutralizing antibodies against sarbecoviruses generated by immunization of macaques with an AS03-adjuvanted COVID-19 vaccine
Source: Sci Transl Med. Author manuscript; Available in PMC 2024 May 10. (PMC11032722; doi:10.1126/scitranslmed.adg7404)
Supplement: Supplemental Material [file NIHMS1980294-supplement-Supplemental_Material.docx]

**MATERIALS AND METHODS**

**Antigen-specific memory B cell (MBC) staining and single-cell sorting**

Banked peripheral blood mononuclear cells (PBMCs) (*10, 11*) were thawed and washed twice with 10 mL of FACS buffer containing 1 x phosphate buffered saline (PBS) plus 2% fetal bovine serum (FBS) and 1 mM EDTA. Samples were then resuspended in 100 μL of 1× PBS containing Zombie ultraviolet (UV) live/dead dye at 1:200 dilution (BioLegend, 423108) and incubated at room temperature for 15 minutes. Following washing, cells were incubated with an antibody cocktail for 1 hour protected from light on ice. The following antibodies were used: CD3 brilliant violet (BV) 650 (BD Biosciences, 563916), CD14 BV650 (BioLegend, 301836), CD16 BV650 (BioLegend, 302042), CD20 allophycocyanin (APC)-cyanine (Cy) 7 (BioLegend, 302314), CD27 phycoerythrin (PE)-Cy7 (BioLegend, 302838), IgM Peridinin-Chlorophyll-Protein (PerCP)-Cy5.5 (BioLegend, 314512), IgD PE (Southern Biotech, 2030-09), IgG brilliant ultraviolet (BUV) 395 (BD Biosciences, 564229) and Alexa Fluor (AF) 488-labeled receptor binding domain (RBD) of SARS-CoV-2 beta variant (SinoBiological, 40592-V08H85-B) and BV421-labeled spike protein (SinoBiological, 40589-V27B-B) or RBD (BioLegend, 793906) protein of SARS-CoV-2 Wuhan strain. All antibodies were used as per the manufacturer's instruction and the final concentration of each probe was 0.1 μg/mL. Single live CD3^-^ CD14^-^ CD16^-^ CD20^+^ IgM^-^ IgD^-^ IgG^+^ Probe^+^ B cells were sorted into individual wells of 96-well plates containing 16 μL of lysis buffer per well using a FACS Aria III and FACSDiva software (Becton Dickinson) for acquisition and FlowJo software (BD Biosciences) for analysis. The sorted cells were immediately frozen on dry ice and used for subsequent RNA reverse transcription as described below.

**Generation of recombinant humanized monoclonal antibodies (mAbs)**

mAbs were generated following established protocols (*60, 61*). In brief, single MBCs were sorted with BD FACS Aria II into 96-well plates containing 16 μL of lysis buffer. The lysis buffer was composed of 20 U RNAse inhibitor (Invitrogen), 5 mM DTT (Invitrogen), 4 μL 5x RT buffer (Invitrogen), 0.0625 μL Igepal (Sigma Aldrich), and 10 μg/mL Carrier RNA (Applied Biosystems). The 96-well plates went through a quick freeze-thaw cycle, and 0.5 μg Oligo(dT)18 (Thermo Fisher Scientific), 0.5 mM dNTP mix (Invitrogen), and 200 U Superscript IV (Invitrogen) was added in a total volume of 4 μL followed by thorough mixing and spinning. The reverse transcription was performed as follows: 10 minutes at 42 ºC, 10 minutes at 23 ºC, 20 minutes at 50 ºC, 5 minutes at 55 ºC, 10 minutes at 80 ºC and finally cooling to 4 ºC. Ig heavy chain and light chain (kappa/lambda) rearrangements were amplified by nested polymerase chain reaction (PCR) using primer cocktails (**table S2**) specific for all V gene families and constant domains at a concentration of 250 nM per primer. The PCR mix consisted of 2.5 μL 10x PCR buffer, 0.5 μl 10 mM dNTP mix (Invitrogen), 0.5 μl 25 mM MgCl_2_ (only added in the first round PCR), 5 µL Q-solution, 1 U HotStarTaq (Qiagen), 0.5 µL 5’ and 3’ primers. Water was added up to a total volume of 25 μL. The PCR programs are shown in **table S3**. The second round PCR products were evaluated on 2% agarose gels, purified using QIAquick spin columns (Qiagen) and sequenced using second round PCR reverse primers. The sequences were analyzed using the online IMGT/HighV-QUEST tool. The productive heavy-light paired Ig genes were used for mAb production (Sino Biological).

**Mesoscale Discovery (MSD) electrochemiluminescence immunoassay (ECLIA)**

V-plex SARS-CoV-2 Panel 9 (human IgG) kit from MSD (K15448U-2) was used to evaluate mAb binding to RBD antigens from the following lineages: A (wild-type), (B.1.1.7), (B.1.214.2), (B.1.351), (B.1.427), (B.1.429), (B.1.525), (B.1.526), (B.1.526.1) (B.1.526.2), (B.1.617), (B.1.617.1), (B.1.617.3), (P.1), (P.3), and (R.1). The assay was performed as per the manufacturer’s instructions. Briefly, the multi-spot 96-well plates were blocked in 0.15 mL of blocking solution with shaking at 700 rpm at room temperature. After 30 minutes of blocking, 50 μL of mAbs was added to each plate in the designated wells and incubated at room temperature for 2 hours with shaking. mAbs were assayed at 10 μg/mL starting concentration and 11 additional 4-fold serial dilutions. Plates were washed and 50 μL of Sulfo-tag conjugated anti-IgG was added, and the plates were incubated at room temperature for 1 hour. After incubation, the plates were washed, and 0.15 mL of MSD-Gold read buffer was added. The plates were immediately read using the MSD instrument (MESO QuickPlex SQ 120MM). The binding capacities of mAbs were presented as the MSD arbitrary light unit areas under the curve (AUC) values, calculated using Prism v9.3.1 (GraphPad).

**Enzyme-linked immunosorbent assay (ELISA)**

All mAbs generated in this study were screened for their binding to the spike proteins of SARS-CoV-2 Wuhan, BA.1, and BA.4/5 strains by ELISA. Briefly, 96-well plates were coated with 50 μL per well of a 2 μg/mL spike protein solution in PBS overnight at 4 °C. Plates were washed 3 times with washing buffer (1× PBS with 0.05% Tween-20 (MP Biomedicals)) and incubated with 200 μL per well blocking buffer (1× PBS with 5% skim milk powder (Bio-Rad) and 0.05% Tween-20 (MP Biomedicals)) for 1 hour at room temperature. Immediately after blocking, 100 μL of each mAb was added to each plate in the designated wells and incubated at room temperature for 1 hour. mAbs were assayed at 1 μg/mL starting concentration and 5 additional 5-fold serial dilutions. Plates were washed and 100 μL of horseradish peroxidase (HRP) conjugated goat anti-human IgG (Sigma, AP112P) in blocking buffer at a 1:5000 dilution was added, and the plates were incubated at room temperature for 1 hour. After incubation, the plates were washed three times and 100 μL per well of 3,3′,5,5′-tetramethybenzidine (Sigma Aldrich, ES022-500ML) was added. The reaction was developed for 4 minutes and stopped by adding 100 μL per well of 450 nm stop solution (Abcam, ab171529). The plates were immediately read using an ELISA microplate reader (Bio-Rad). The binding capacities of mAbs were presented as ELISA AUC values, calculated using Prism v9.3.1 (GraphPad).

For experiments evaluating the competition between mAbs for RBD binding, purified mAbs (CR3022, CC12.3, CV07-270, S2X259, and S2M11) were biotinylated using the Lightning-Link Biotinylation Kit (Abcam, ab201795) according to the manufacturer’s instructions. 96-well plates were coated with 50 μL per well of a 2 μg/ml spike protein solution in PBS overnight at 4 °C. Plates were washed 3 times with washing buffer (1× PBS with 0.05% Tween-20 (MP Biomedicals)) and incubated with 200 μL per well blocking buffer (1× PBS with 5% skim milk powder (Bio-Rad) and 0.05% Tween-20 (MP Biomedicals)) for 1 hour at room temperature Immediately after blocking, 100 μL of mAbs was added to each plate in the designated wells and incubated at room temperature for 30 minutes. mAbs were assayed at 10 μg/mL starting concentration and 6 additional two-fold serial dilutions. Then, without washing, 100 μL of biotinylated mAbs at 500 ng/mL was added and incubated for additional 30 minutes, followed by detection using HRP-conjugated streptavidin (Abcam, ab7403) and TMB (Sigma Aldrich, ES022-500ML). Background by the HRP-conjugated detection antibodies alone was subtracted from all absorbance values.

**Pseudovirus neutralization assay (PsVNA)**

For initial screening, neutralization assays using pseudotyped viruses (using HIV backbone) carrying spike proteins of SARS-CoV-2 wild type, BA.1, and BA.4/5 strains, respectively, were performed by Sino Biological Inc. Briefly, 293T cells overexpressing angiotensin converting enzyme 2 (ACE2) were seeded in 96-well plates (Costar, 3955) at 3 × 10^4^ cells per well. Fifty μL of serially-diluted mAbs or a control mAb (S2H97) were mixed with 50 μl of pseudovirus and then they were added into plates seeded with 293T-ACE2 cells (1 × 10^4^ half-maximal tissue culture infectious dose (TCID_50_)/mL). A positive control was set up using a mixture of 50 μL Dulbecco’s modified Eagle’s medium (DMEM) and 50 μL pseudovirus. One hundred μL DMEM was used as a negative control. Cells were cultured at 37 °C for 64 hours. Luminous value was detected by Luminometer (Berthold Technologies, Centro LB 960) as relatively light units (RLUs) and the inhibitory rate was calculated by (1 − (mean RLU of sample − RLU of negative control) / (RLU of positive control − RLU of negative control) *100%). Experiments were performed in duplicate.

The 15 most potent BA.1 neutralizing mAbs were evaluated again in a qualified SARS-CoV-2 PsVNA performed in the laboratory of U.S. Food and Drug Administration. Pseudovirions were produced as previously described (*62,63*). Briefly, human codon-optimized cDNA encoding SARS-CoV-2 spike protein of the WA-1/2020 and variants and SARS-CoV spike protein were synthesized by GenScript and cloned into eukaryotic cell expression vector pcDNA 3.1 between the BamHI and XhoI sites. Pseudovirions were produced by co-transfection Lenti‐X 293T cells with psPAX2(gag/pol), pTrip-luc lentiviral vector and pcDNA 3.1 SARS-CoV-2-spike-deltaC19, using Lipofectamine 3000. The supernatants were harvested at 48 hours post-transfection, filtered through 0.45µm membranes, and titrated using 293T-ACE2-TMPRSS2 cells (HEK 293T cells expressing ACE2 and transmembrane serine protease 2 proteins).

Neutralization assays were performed as previously described (*64, 65*). For the neutralization assay, 50 µL of pseudovirions (counting about 200,000 relative light units) were pre-incubated with an equal volume of fresh minimum essential medium containing serial dilutions of mAbs at room temperature for 1 hour. Then, 50 µL of virus and mAb or serum mixtures were added to 293T-ACE2-TMPRSS2 cells (10^4^ cells/50 μL) in triplicates in a 96-well plate. Controls included a cells-only control and a virus without any antibody control. After a 3 hour incubation, fresh medium was added to the wells. Cells were lysed 24 hours later, and luciferase activity was measured using One-Glo luciferase assay system (Promega, E6130). Each assay was performed in duplicate, and the 50% neutralization titer was calculated using Prism 9 (GraphPad Software). Generation of vesicular stomatitis virus (VSV) pseudovirus and VSV pseudovirus entry assays were performed as previously described (*20*).

**Production of Viruses for in vitro neutralization and in vivo challenge assays**

All recombinant infectious clone-based viruses were approved by the University of North Carolina at Chapel Hill Institutional Review Board under Schedule G 78684, 100475, 60350, 104615, and 11515. Viruses were designed using our previously described infectious clone systems to incorporate mutations that were previously defined as necessary for effective pathogenic infection of mice. These mouse-adapted mutations (MA) were used for the design of the SARS-CoV-2 in vivo challenge viruses (MA10), as well as for our SARS-CoV and SHC014 MA15 viruses. Additionally, viruses used in live virus neutralization assays were derived from infectious clones where the *ORF7* gene was replaced with a nano-luciferase gene cassette.

Viruses were derived following the ligation of cDNA of infections clone fragments, followed by in vitro transcription with mMessage Machine T7 polymerase (Thermo Fisher). A separate reaction using a T7 promoter upstream of the nucleocapsid gene was used to produce nucleocapsid mRNA to aid in virus replication and recovery. Prior to electroporation, cells were mixed with the full-length mRNA as well as the nucleocapsid mRNA. Vero E6 cells over-expressing human TMPRSS2 and ACE2 were electroporated in PBS (Gibco) under the following conditions: 450 volts, 50 microfarads, 4 pulses and allowed to recover for 10 minutes. The cells were then plated into a T75 flask. Passage 0 (p0) stocks were recovered about 24 to 36 hours post-electroporation. Passage 1 working stocks were created by inoculation of a confluent T175 flask of Vero E6 TMPRSS2-ACE2 cells with 1 mL of p0 virus. Stocks were then titered by plaque assay where virus was serially diluted ten-fold and inoculated onto confluent monolayers of Vero E6 cells in 6-well plates. Plates were incubated for 1 hour with gentle rocking every 15 minutes. Subsequently, DMEM (Gibco) with Fetal Clone II serum (FCII, Hyclone), and 1× antibiotic/antimycotic (Gibco), and 0.8% agarose was applied as an overlay. Three days later, resulting plaques were visualized via staining with 0.05% neutral red dye (Chem-Impex International) in PBS (Gibco).

**Live-virus neutralization assays**

Antibodies were diluted 1:20 by adding 11.25 μL of antibodies to 213.75 μLDMEM (Gibco) supplemented with 10% FCII. Antibodies were then serially diluted 1:3 seven times in triplicates by adding 75 μL of the previous dilution into 150 μL of media with 75 μL removed from the final well. Next, 150 μL of media containing 1600 plaque-forming units (PFU)/mL of viruses expressing nanoluciferase (nLuc) was mixed with the diluted antibodies and allowed to incubate for 1 hour at 37℃, after which 100 μL of the virus-antibody mix was added to individual wells in a 96-well plate seeded 24 hours prior with 2x10^4^ cells, for a final 800 PFU of virus per well. Plates were incubated for 48 hours at 37℃ with 5% CO_2_. After incubation, luciferase activity was measured with the Nano-Glo Luciferase Assay System (Promega) according to the manufacturer specifications. Neutralization titers (IC_50_) were defined as the dilution at which a 50% reduction in RLU was observed relative to the virus (no antibody) control.

**In vivo challenge experiments**

All animal work was approved by Institutional Animal Care and Use Committee at University of North Carolina at Chapel Hill under protocol 20-114 and 20-200 according to guidelines outlined by the Association for the Assessment and Accreditation of Laboratory Animal Care and the U.S. Department of Agriculture. All virus studies were performed in animal biosafety level 3 facilities at University of North Carolina at Chapel Hill.

Female BALB/c mice were obtained from Envigo (strain 047). Twelve hours prior to infection, mice received 200 μg of indicated mAbs or an isotype recombinant human anti-Dengue envelope antibody (2D22) intraperitoneally. The following morning, mice were infected intranasally under ketamine/xylazine anesthesia with PFU doses of 1x10^3^ (SARS-CoV-2 MA10), 1x10^4^ (SARS-CoV MA15), or 1x10^5^ (SARS-CoV-2 BA.1 and SARS-CoV MA15 SHC014) in 50 μL of PBS (Gibco). At indicated timepoints, a subset of mice were euthanized by isoflurane overdose, and lung tissue was harvested for quantification of viral titers, evaluation of gross lung discoloration, and histopathological analyses. Titer and RNA samples were stored at −80°C, and histopathology samples were stored at 4°C in 10% phosphate buffered formalin Gross lung discoloration scores were based on the number and severity of visible lung surface hemorrhages at the time of harvest (dark red versus anatomical pink coloring).

**Biolayer Interferometry (BLI) Binding Assay**

RBD proteins for the BLI binding assay were expressed in human cells. RBDs were cloned into phCMV3 vector and fused with a C-terminal His_6_ tag. The plasmids were transiently transfected into Expi293F cells using ExpiFectamine 293 reagent (Thermo Fisher Scientific) according to the manufacturer’s instructions. The supernatant was collected at 7 days post-transfection. The His_6_-tagged proteins were then purified with Ni Sepharose Excel protein purification resin (Cytiva) followed by size exclusion chromatography. Omicron RBD was purchased from ACROBiosystems Inc.

The BLI assays were performed using an Octet Red instrument (FortéBio) as described previously (*28*). To measure the binding kinetics of mAbs and RBDs, the mAbs were diluted with kinetic buffer (1× PBS [pH 7.4] plus 0.01% bovine serum albumin and 0.002% Tween-20) into 15 µg/mL. The mAbs were then loaded onto anti-human IgG Fc (AHC) biosensors and interacted with 100 nM of RBDs. The assay consisted of the following steps: 1) baseline, 1 minute with 1× kinetic buffer; 2) loading, 90 seconds with mAbs; 3) wash, 15 seconds wash of unbound mAbs with 1× kinetic buffer; 4) baseline, 1 minute with 1× kinetic buffer; 5) association, 90 seconds with RBDs; and 6) dissociation, 90 seconds with 1× kinetic buffer. For estimating the dissociation constant (K_D_), a 1:1 binding model was used.

**Crystallization and structural determination**

Expression and purification of the SARS-CoV-2 RBDs for crystallization were as described previously (*21*). Briefly, wild-type and BA.2 SARS-CoV-2 RBDs (residues 333-529) of the spike proteins were cloned into a customized pFastBac vector (*66*) and fused with an N-terminal gp67 signal peptide and C-terminal His_6_ tag. The recombinant bacmid DNAs were generated using the Bac-to-Bac system (Life Technologies). Baculoviruses were generated by transfecting purified bacmid DNAs into Sf9 cells using FuGENE HD (Promega), and subsequently used to infect suspension cultures of High Five cells (Life Technologies) at a multiplicity of infection of 5 to 10. Infected High Five cells were incubated at 28 °C with shaking at 110 rpm for 72 hours for protein expression. The supernatants were then concentrated using a 10 kDa molecular weight cutoff Centramate cassette (Pall Corporation). The RBD proteins were purified by Ni-NTA, followed by size exclusion chromatography, and buffer was exchanged into 20 mM Tris-HCl pH 7.4 and 150 mM NaCl.

25F9/RBD (wild-type), 21B6/RBD (wild-type), 20A7/RBD (wild-type), and 20A7/RBD (BA.2) complexes were formed by mixing each of the protein components in an equimolar ratio and incubating overnight at 4°C. The protein complexes were adjusted to 11 to 12 mg/mL and screened for crystallization using the 384 conditions of the JCSG Core Suite (Qiagen) on a robotic CrystalMation system (Rigaku) at Scripps Research. Crystallization trials were set-up by the vapor diffusion method in sitting drops containing 0.1 μL of protein and 0.1 μL of reservoir solution. For the 25F9/RBD (wild-type) complex, optimized crystals were grown in drops containing 1.6 M ammonium sulfate, 0.1 M bicine pH 9, and 15% glycerol at 20°C. Crystals appeared on day 14 and were harvested on day 28 by soaking in reservoir solution supplemented with 15% (v/v) glycerol. Diffraction data were collected at cryogenic temperature (100 K) at beamline 23-ID-D of the Advanced Photon Source (APS) at Argonne National Labs. For the 21B6/RBD (wild-type) complex, optimized crystals were grown in drops containing 0.1 M sodium citrate, pH 3.3 and 1.45 M ammonium sulfate at 20°C. Crystals appeared on day 7 and were harvested on day 15 by soaking in reservoir solution supplemented with 20% (v/v) ethylene glycol. Diffraction data were collected at cryogenic temperature (100 K) at beamline 23-ID-B of the APS at Argonne National Labs. For the 20A7/RBD (wild-type) complex, optimized crystals were grown in drops containing 0.2 M CaCl_2_, 10% ethylene glycol (v/v), and 20% polyethylene glycol 3350 (w/v) at 20°C. Crystals appeared on day 7 and were harvested on day 15 with no additional cryoprotectant. Diffraction data were collected at cryogenic temperature (100 K) at beamline 23-ID-B of the APS at Argonne National Labs. For the 20A7/RBD (BA.2) complex, optimized crystals were then grown in drops containing 0.1 M HEPES pH 7.5, 10% (v/v) glycerol, 5% (w/v) polyethylene glycol 3000, and 30% (v/v) polyethylene glycol 400 at 20°C. Crystals appeared on day 7 and were harvested on day 10 by soaking in reservoir solution supplemented with 15% (v/v) ethylene glycol. Diffraction data were collected at cryogenic temperature (100 K) at the Stanford Synchrotron Radiation Lightsource (SSRL) on Scripps/Stanford beamline 12-1. Diffraction data were processed with HKL2000 (*67*). Structures were solved by molecular replacement using PHASER (*68*). Iterative model building and refinement were carried out in COOT (*69*) and PHENIX (*70*), respectively. Epitope and paratope residues, as well as their interactions, were identified by accessing PISA at the European Bioinformatics Institute (http://www.ebi.ac.uk/pdbe/prot_int/pistart.html) (*71*).

**Supplementary Figures**

**
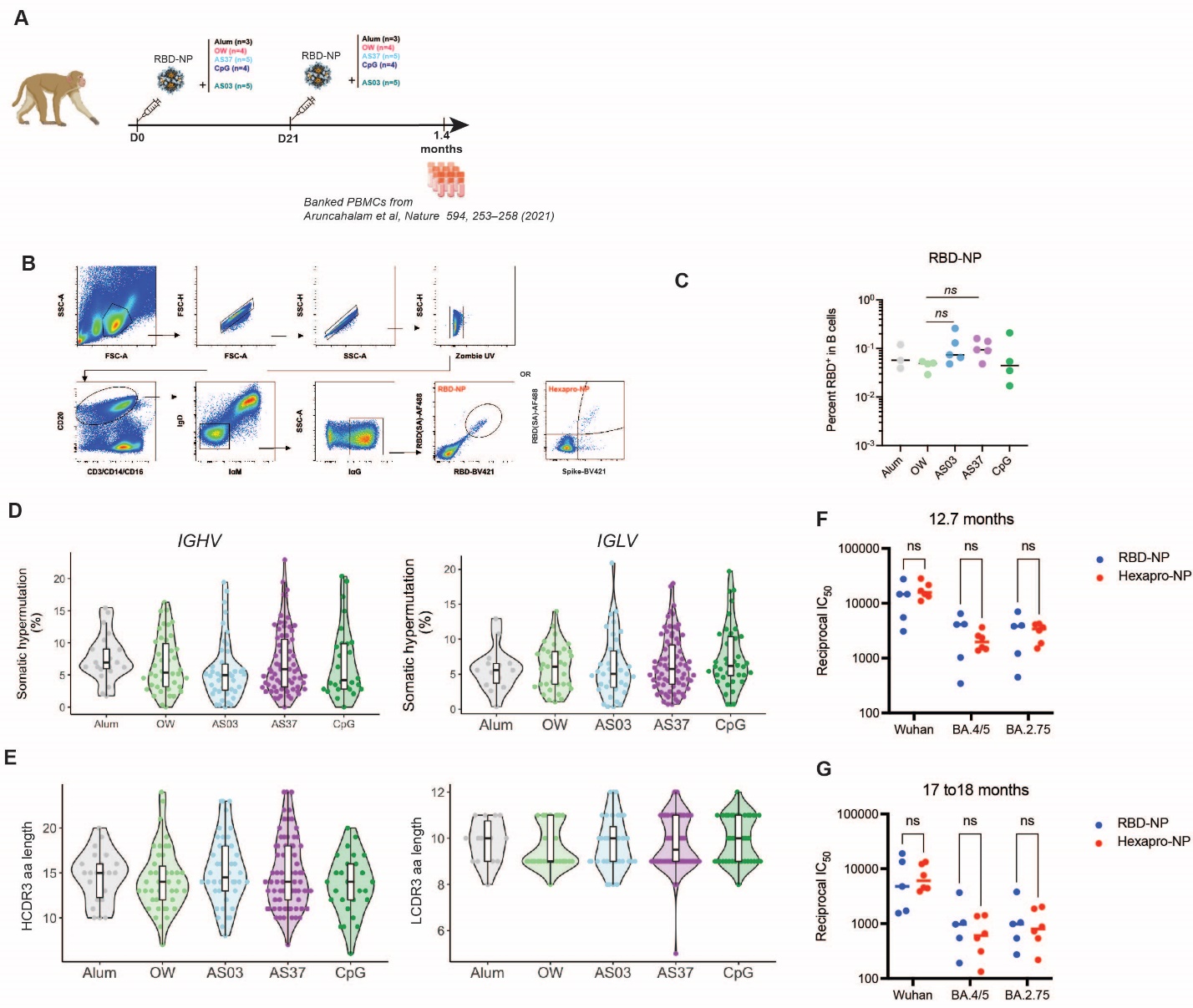
**

**Figure S1. Comparison of MBC responses in different adjuvant groups.**

(**A**) Schematic representation of the study design. (**B**) Shown is the gating strategy used to analyze the percentage of spike^+^ and RBD^+^ B cells of total CD20^+^ cells. Gating was on singlets that were live CD20^+^ CD3^-^ CD14^-^ CD16^-^ IgM^-^/D^-^ IgG^+^ Antigen^+^. SSC, side scatter; FSC, forward scatter; A, area; H, height. (**C**) The dot plot summarizes the percentage of RBD-binding B cells in total CD20^+^ B cells in 21 vaccinated individuals. Horizontal bars indicate mean values. Statistical significance between any two groups was determined using two-tailed Mann–Whitney U tests (ns> 0.05). n = 3 (Alum), n = 5 (AS03 and AS37), and n = 4 (CpG-Alum and OW). (**D**) Graphs show the somatic hypermutation rates of the productive *IGHV* (left) and *IGLV* (right) genes isolated from animals vaccinated with RBD-NP plus different adjuvants at day 42. The boxes inside the violin plot show median, upper, and lower quartiles. Each dot represents an individual gene. For heavy chain, n = 22 (Alum), n = 42 (OW), n = 44 (AS03), n = 74 (AS37), n = 29 (CpG) and for light chain, n = 14 (Alum), n = 35 (OW), n = 37 (AS03), n = 78 (AS37), n = 40 (CpG). (**E**) as in (D), but for complementarity determining region (CDR) 3 length in amino acids (aa). The boxes inside the violin plot show median, upper, and lower quartiles. Each dot represents an individual gene. For heavy chain, n = 22 (Alum), n = 42 (OW), n = 44 (AS03), n = 74 (AS37), n = 29 (CpG) and for light chain, n = 14 (Alum), n = 35 (OW), n = 37 (AS03), n = 78 (AS37), n = 40 (CpG). (**F**) Pseudovirus neutralizing antibody responses are shown for samples collected at 12.7 months against viruses indicated on X-axis are shown. Each symbol represents an animal [RBD-NP (blue; n = 5) and Hexapro-NP (red; n = 6)]. The statistical differences were calculated using two-way ANOVA (ns> 0.05). (**G**) Pseudovirus neutralizing antibody responses are shown for samples collected at 17 to 18 months against viruses indicated on X-axis are shown. Each symbol represents an animal [RBD-NP (blue; n = 5) and Hexapro-NP (red; n = 6)]. The statistical differences were calculated using two-way ANOVA (ns> 0.05).


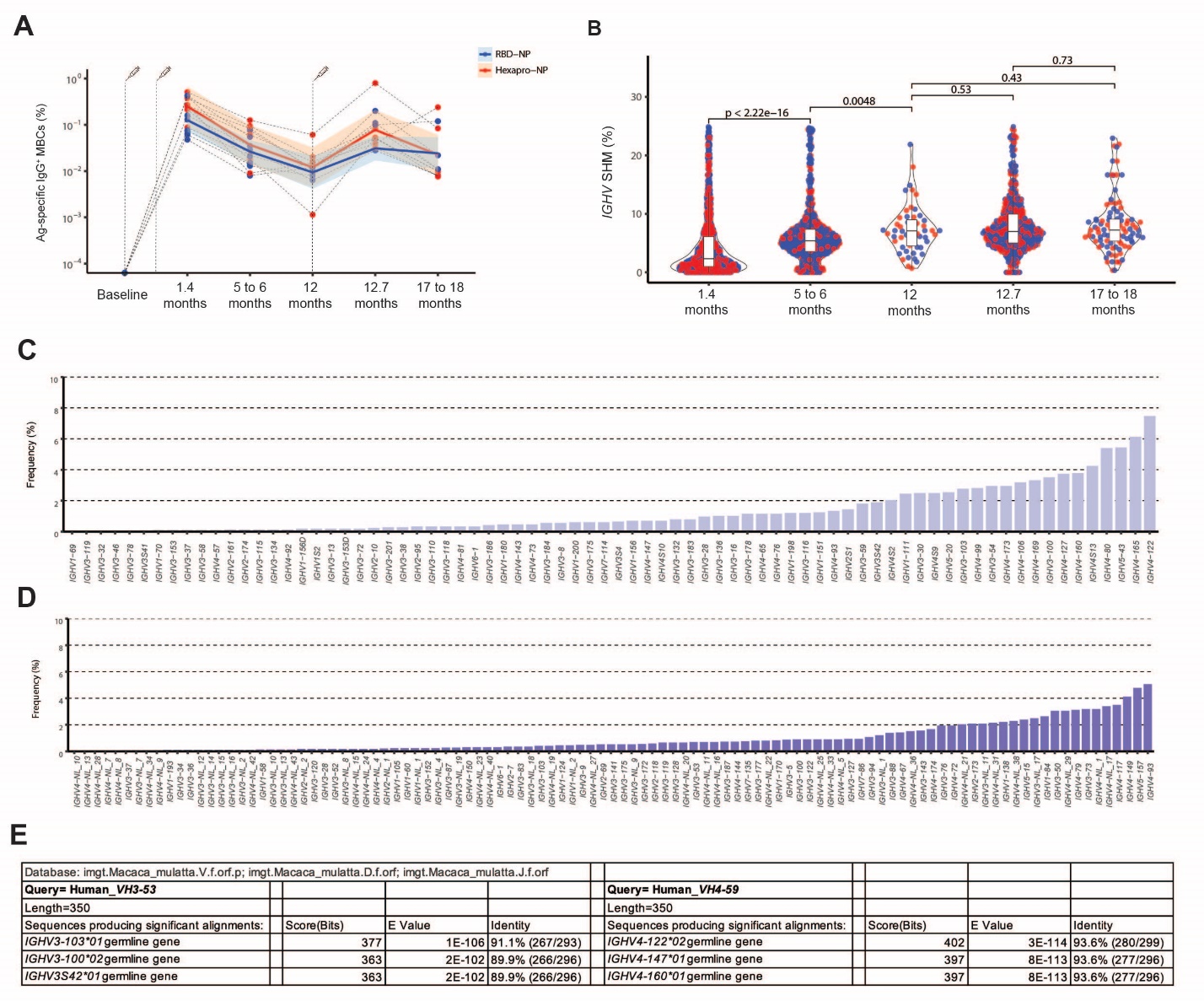


**Figure S2. Antibody and MBC responses.**

(**A**) Frequency of antigen (spike protein- or RBD-; Ag)-specific MBCs relative to CD20^+^ B cells is shown for samples from RBD-NP (blue) or Hexapro-NP (red) group. Paired samples are connected with a dashed line. Geometric means are presented in thick lines, and shades indicate 95% confidence intervals. (**B**) Shown is SHM analysis using KIMDB *IGHV* database. Somatic hypermutation rates are shown for the productive *IGHV* genes of B cells isolated from RBD-NP (blue) or Hexapro-NP (red) vaccinated animals at indicated time points. The boxes inside the violin plot show median, upper, and lower quartiles. Each dot represents an individual gene. The statistical differences between timepoints were calculated using one-way ANOVA. (**C**) The graph shows the relative abundance of *IGVH* gene usage in antigen-specific MBCs in Rhesus macaques calculated using the IMGT database. (**D**) The graph shows the relative abundance of *IGVH* gene usage in antigen-specific memory B cells in Rhesus macaques calculated using the KIMDB database. (**E**) Shown is IgBlast analysis of human *VH3-53* germline gene and human *VH4-59* germline gene using the IGMT rhesus macaque database.


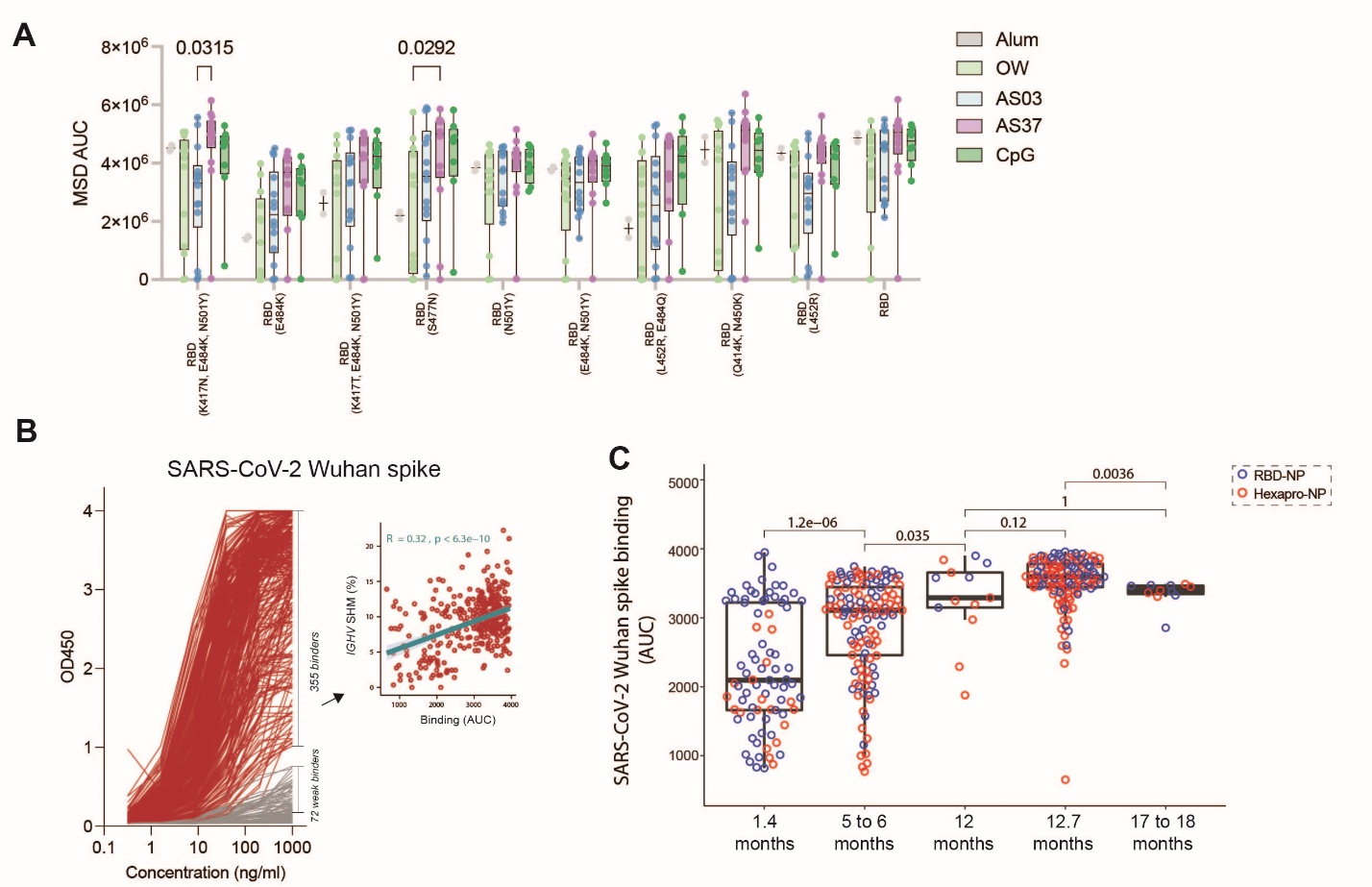


**Figure S3. Binding profiles of mAbs.**

(**A**) Graphs show results of MSD ECLIAs measuring cross-reactive binding profiles of mAbs isolated from individuals vaccinated with RBD-NP plus different adjuvants. Data are presented as areas under the curve (AUC). Black horizontal bars represent the median. Statistical significance was determined using a one-way ANOVA with Tukey’s multiple comparisons test. Significant P values are indicated. OW: Essai O/W 1849101 (a squalene-in-water emulsion). (**B**) Graphs show anti-SARS-CoV-2 Wuhan spike protein ELISA titration curves for all 514 mAbs generated in this study. mAbs showing optical density (OD) 450 above 1.0 at 1 μg/mL concentration were highlighted in red. The Spearman’s correlation between the binding AUC and somatic hypermutation rates (SHM) of *IGHV* genes of the highlighted antibodies is shown on the right. (**C**) Graphs show the anti-SARS-CoV-2 Wuhan spike protein activity (AUC) of the highlighted mAbs in (B) at indicated time points. The boxes show the median, upper, and lower quartiles. The whiskers show min to max. Each dot represents one antibody. The statistical differences between time points were calculated using one-way ANOVA. P values are indicated.


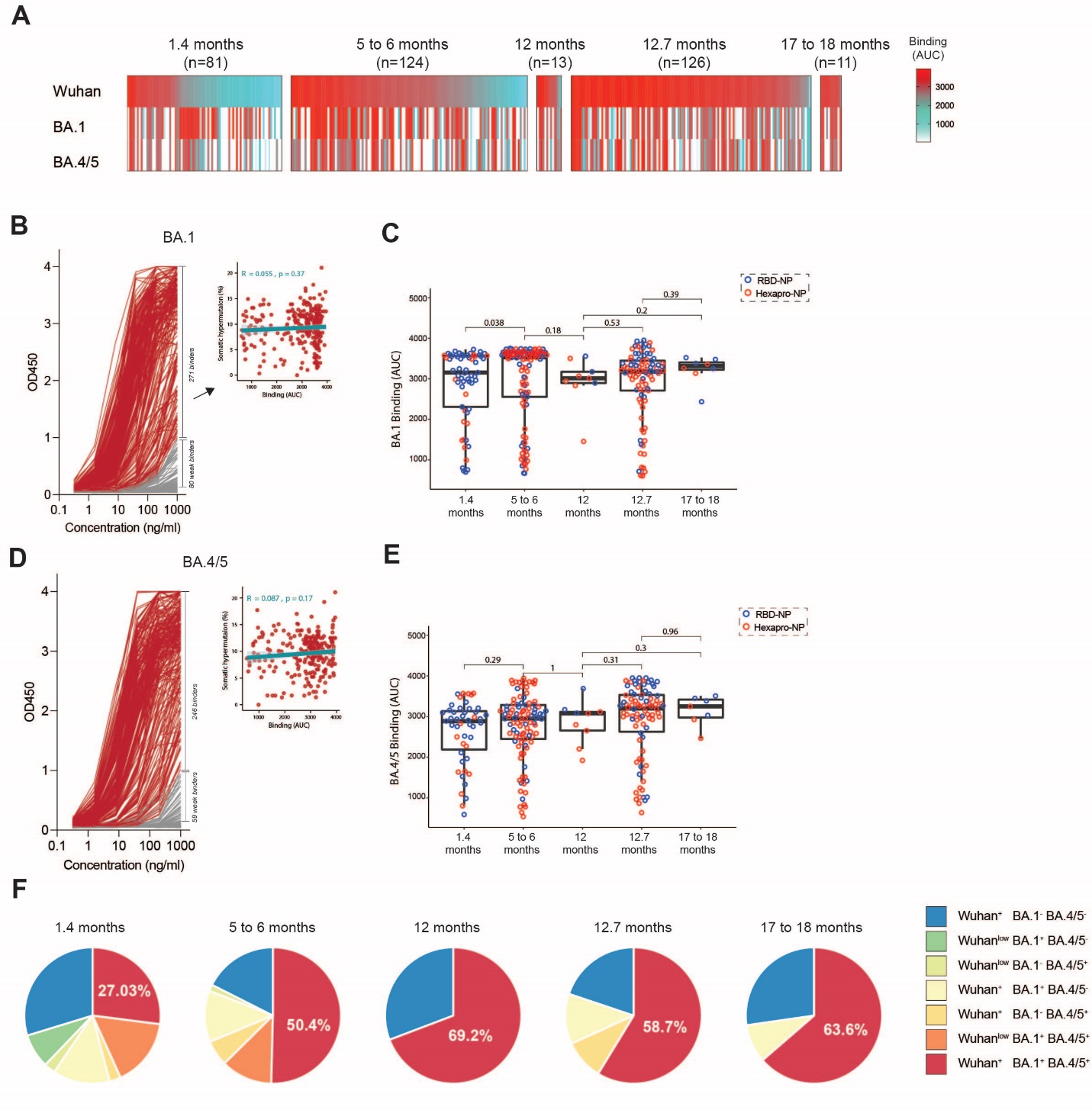


**Figure S4. Cross-reactive properties of mAbs.**

1. Heatmaps show the binding activities of the red highlighted antibodies in fig. S3B against

Spike proteins of SARS-CoV-2 Wuhan, BA.1, and BA.4/5. Antibodies isolated at indicated time points were grouped (the number of antibodies per group was shown in brackets). The gradient color bar indicated the binding capacity (AUC) from 40 to 4000. (**B**) Graphs show anti-SARS-CoV-2 BA.1 spike protein ELISA titration curves for all 514 mAbs generated in this study. mAbs showing OD450 above 1.0 at 1 μg/mL concentration were highlighted in red. The Spearman’s correlation between the BA.1 binding AUC and somatic hypermutation rates (SHM) of *IGHV* genes of the highlighted mAbs is shown on the right. (**C**) Graphs show the anti-SARS-CoV-2 BA.1 spike protein activity (AUC) of the highlighted mAbs in (B) at indicated time points. (**D and E**) As in (B) and (C), but showing ELISA titration curves (**D**) and antibody activity (**E**) against SARS-CoV-2 BA.4/5. In (C) and (E), the boxes show the median, upper, and lower quartiles, and the whiskers show min to max. Each dot represents an antibody. The statistical differences between time points were calculated using one-way ANOVA. P values are indicated. (**F**) The antibodies were categorized based on the cross-reactive properties as shown in the right legends. The pie charts illustrate the fraction of antibodies in each category at indicated time points, and the number of antibodies per group was the same as in (A).


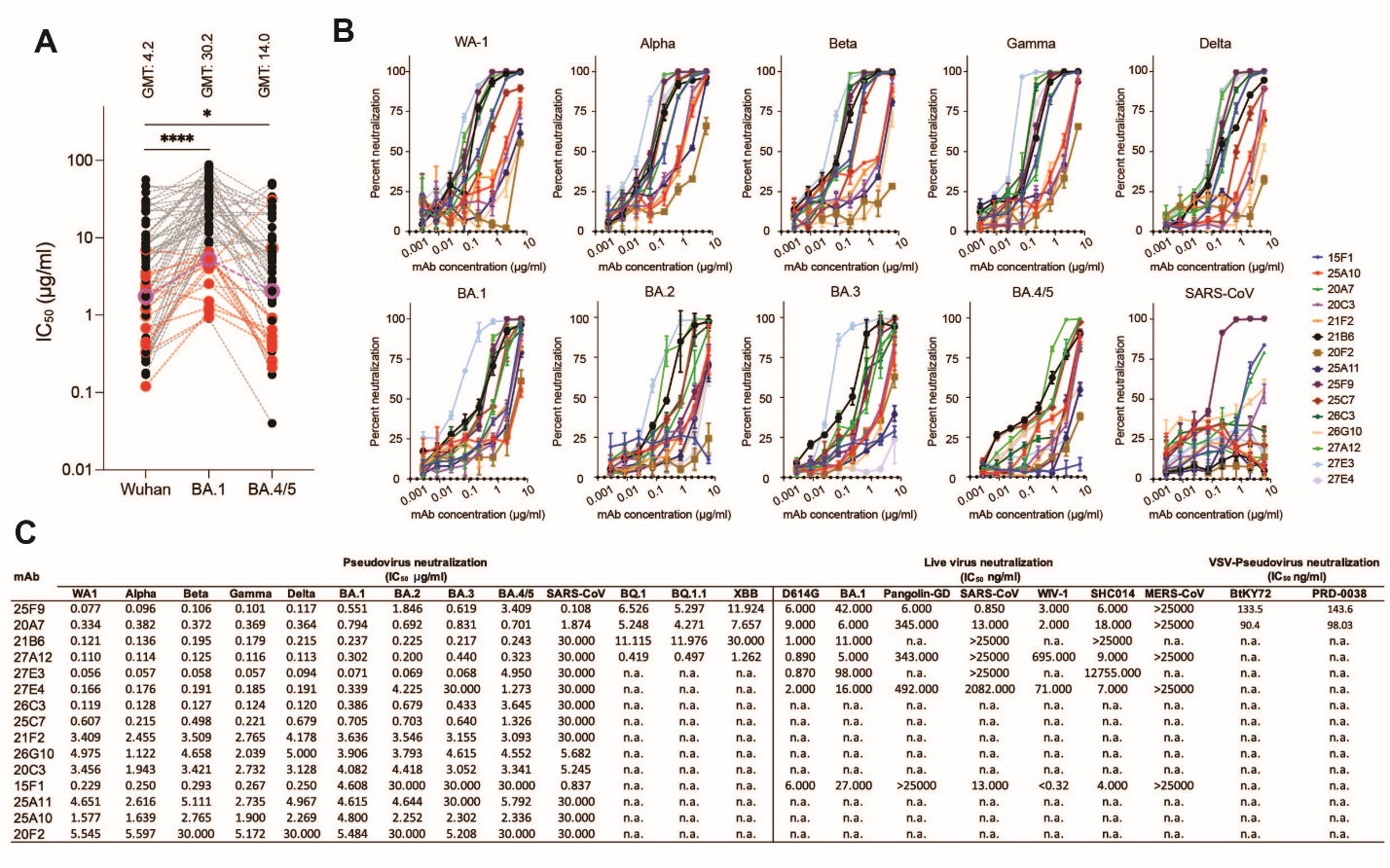


**Figure S5. Binding and neutralizing activities of the top 15 SARS-CoV-2 BA.1 neutralizing mAbs.**

(**A**) mAbs showing comparable or better SARS-CoV-2 BA.1 neutralizing potency (lower IC_50_, µg/ml) than that of a control antibody, S2H97 (purple) were highlighted in red and selected for further characterization. The numbers within the graphs show geometric mean titers (GMTs). The statistical differences between viruses were calculated using a one-way ANOVA (*P < 0.05, ****P < 0.0001). (**B**) Shown is the neutralization capacity of 15 mAbs against pseudotyped SARS-CoV-2 (WA1), 4 previous SARS-CoV-2 variants of concern [B.1.1.7 (Alpha), B.1.351 (Beta), P.1 (Gamma), B.1.617.2 (Delta)], current variants of concern SARS-CoV-2 Omicron sublineage (BA.1, BA.2, BA.3, BA.4/5) and SARS-CoV. Data are presented as the mean ± standard deviation. n = 3 replicates per antibody. (**C**) Shown is a summary of mAb neutralization of pseudotyped SARS-related viruses and authentic SARS-CoV-2 D614G, Omicron BA.1, and Pangolin, SARS-CoV, WIV1, SHC014, and MERS-CoV. Data are presented as IC_50_ values. n.a. represents not available.

**
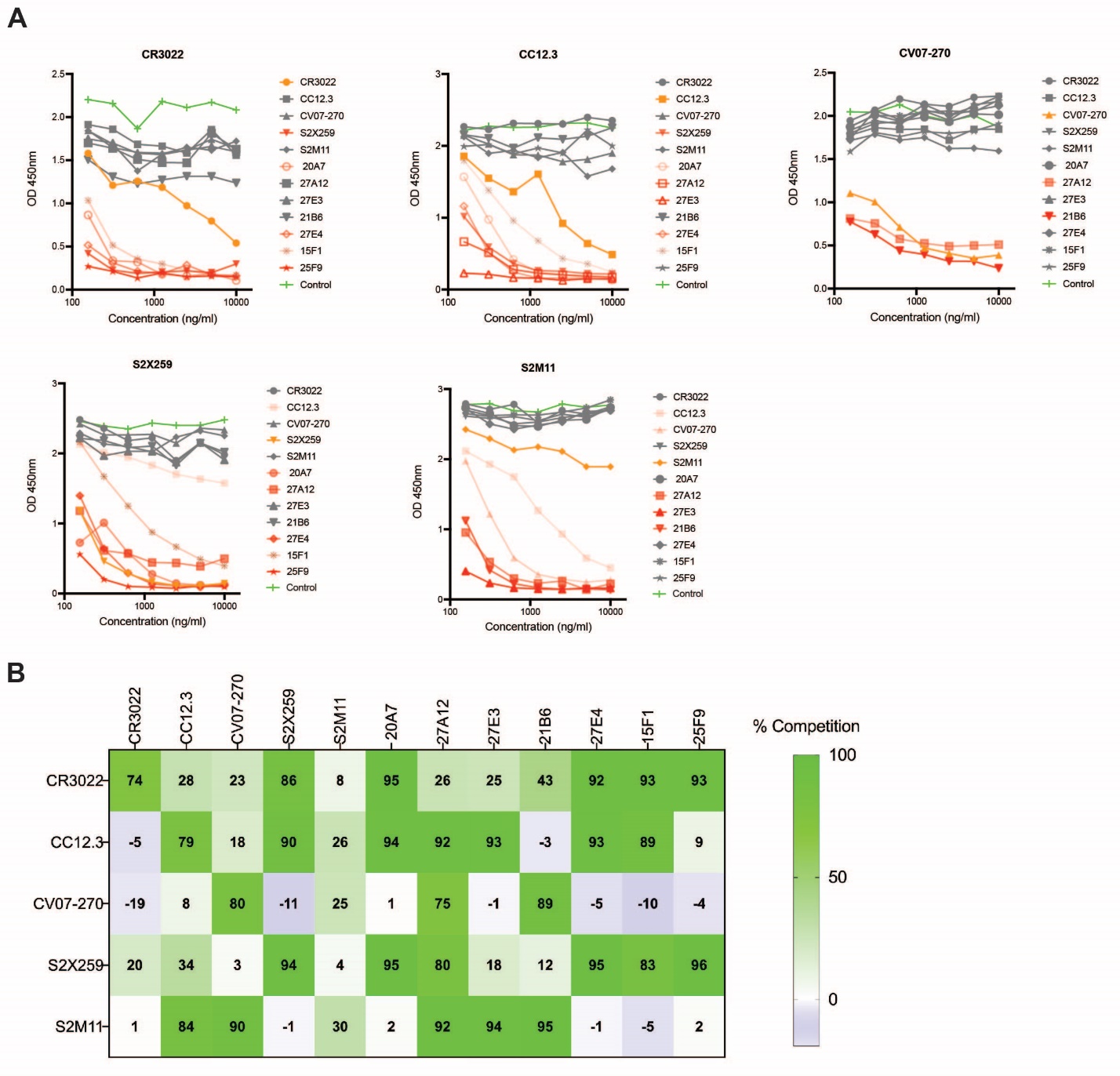
**

**Figure S6. Binding Epitope Characterization of the 7 selected mAbs.**

(**A**) Shown are the results of a competition ELISA (blockade-of-binding) between selected mAbs (labeled in different shapes and colors) at serially diluted concentrations and the indicated biotinylated antibodies (shown as the title) with known binding epitopes at a fixed concentration of 0.5 μg/mL. Controls (green) show the absorbance values with only the detection antibody. (**B**) The heatmap illustrates the competition for SARS-CoV-2 spike protein binding between combinations of selected mAbs. Shades and percentages in squares indicate the degree of competition for spike protein binding of detection antibodies (row) in the presence of mAbs (column) at a concentration of 10 μg/mL.


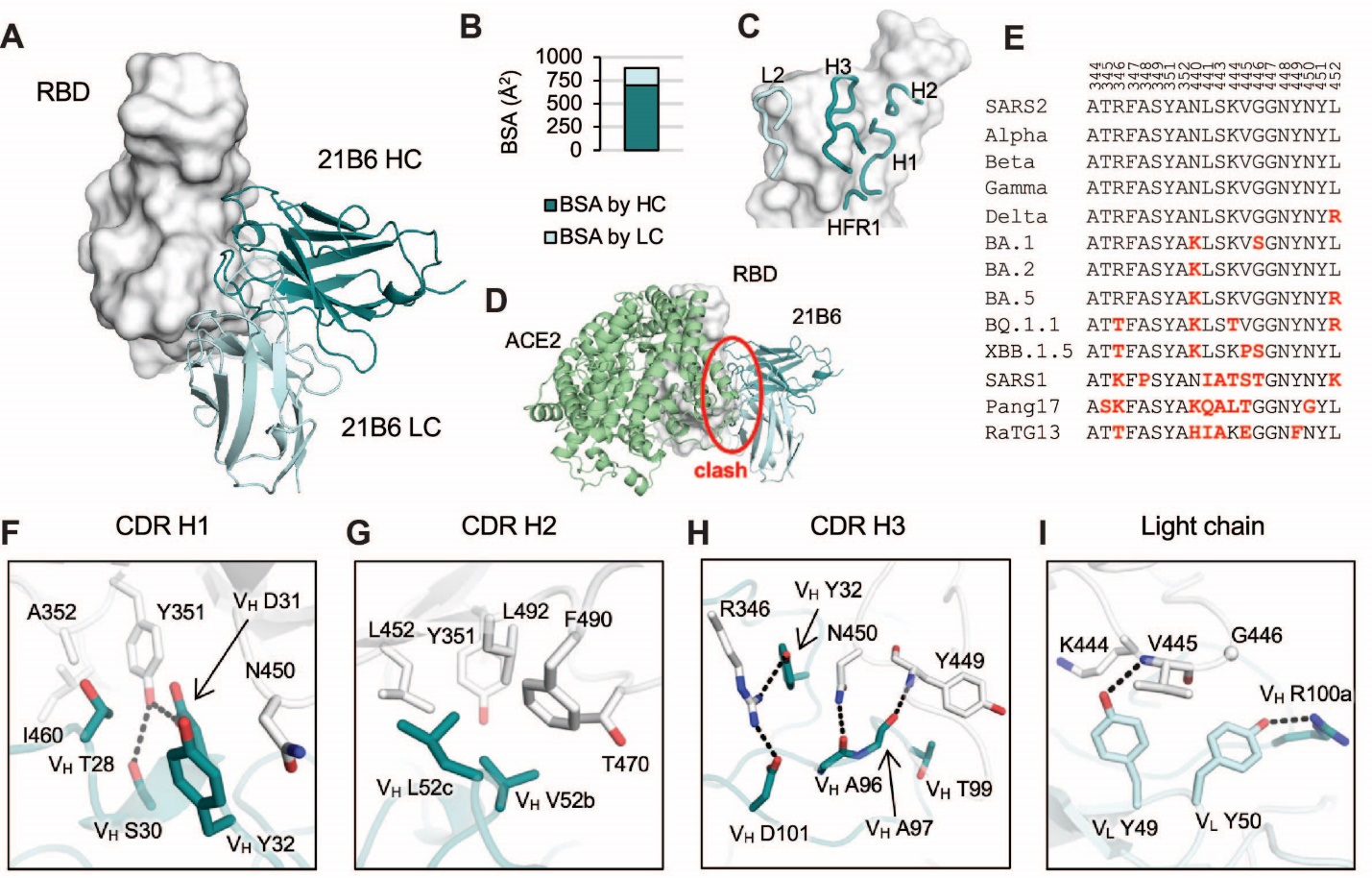


**Figure S7. Crystal structure of 21B6 in complex with SARS-CoV-2 RBD.**

The heavy and light chains of 21B6 variable domains are in dark and light teal. (**A**) Shown is the crystal structure of 21B6 in complex with SARS-CoV-2 RBD. (**B**) Shown is the surface area of SARS-CoV-2 buried (buried surface area or BSA) by heavy and light chains of 21B6. (**C**) 21B6 interacts with RBD with heavy (H) and light (L) chain CDRs H1, H2, H3, L2, and heavy chain framework region (HFR) 1. (**D**) SARS-CoV-2 RBD in complex with 21B6 superimposed onto an RBD-ACE2 complex structure (PDB 6M0J) shows that 21B6 would clash (red circle) with ACE2. (**E**) Shown is sequence alignment of epitope residues (defined as BSA > 0 Å^2^) in a subset of SARS-like viruses. Residues that differ from wild-type SARS-CoV-2 are indicated in red. (**F to I**) Molecular interactions between RBD and 21B6 are shown. Interactions with CDR H1 (**F**), CDR H2 (**G**), CDR H3 (**H**), and light chain (**I**) are presented. Hydrogen bonds and salt bridges are indicated by dashed lines.


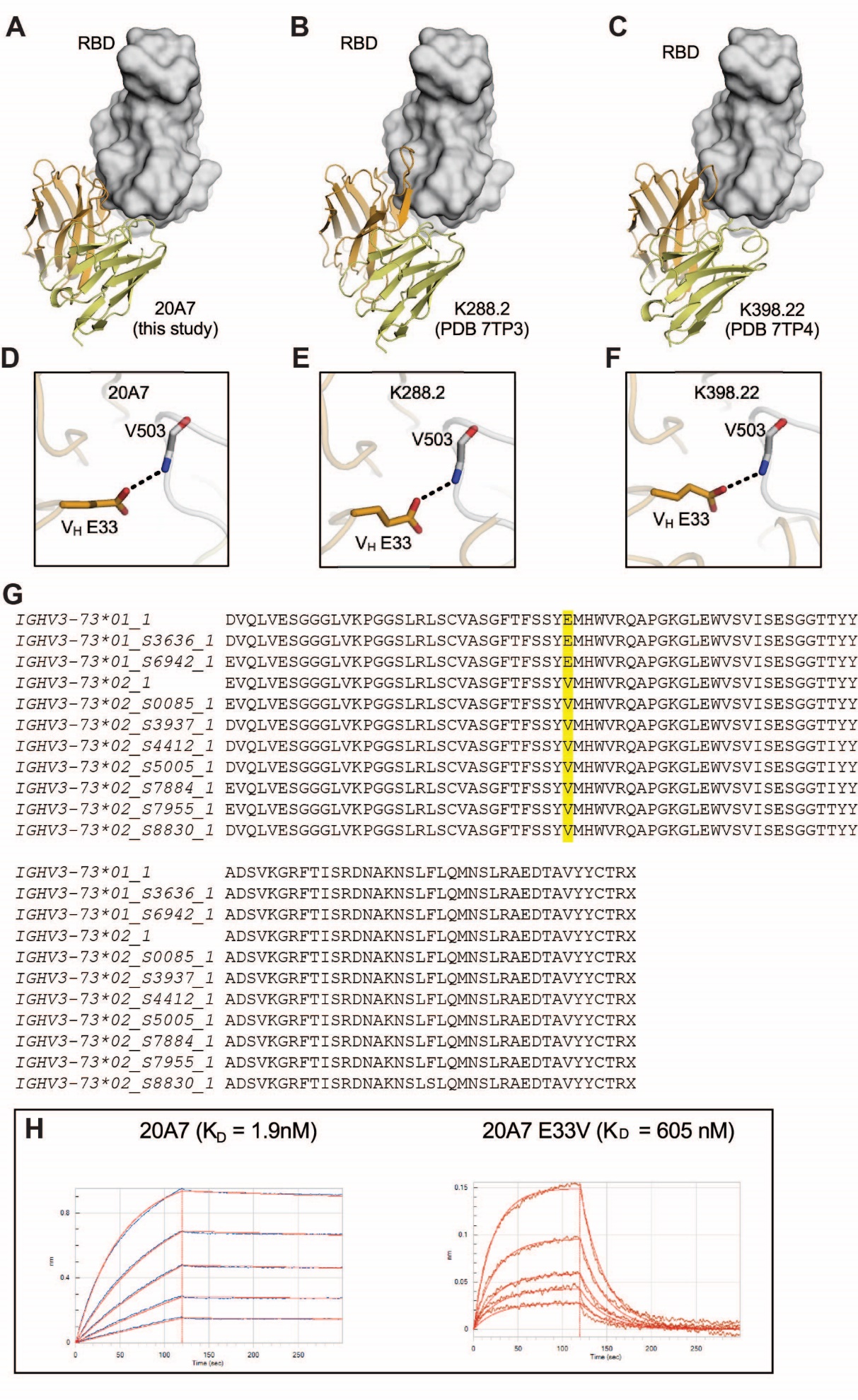


**Figure S8. Structural convergence of rhesus macaque *IGHV3-73*-encoded antibodies targeting the SARS-CoV-2 RBD.**

SARS-CoV-2 RBDs are shown in whitish grey, and the antibody heavy and light chains are shown in orange and yellow. Hydrogen bonds and salt bridges are indicated by dashed lines. (**A to C**) Rhesus macaque *IGHV3-73*-encoded antibody 20A7 (**A**) adopts the same binding mode as two previously published antibodies, K288.2 (**B**) and K398.22 (**C**), encoded by the same VH germline gene (*22*). (**D to F**) Residue V_H_ E33 of all three *IGHV3-73*-encoded antibodies, 20A7 (**D**), K288.2 (**E**), and 398.22 (**F**), form a hydrogen bond with the backbone amide of RBD-V503. (**G**) Shown is sequence alignment of all *IGHV3-73* alleles available in the macaque KIMDB *Ig* database. Residues at position 33 are highlighted in yellow. (**H**) Shown are results of a BLI assay of 20A7 wild-type Fab and an E33V mutant binding to SARS-CoV-2 RBD.


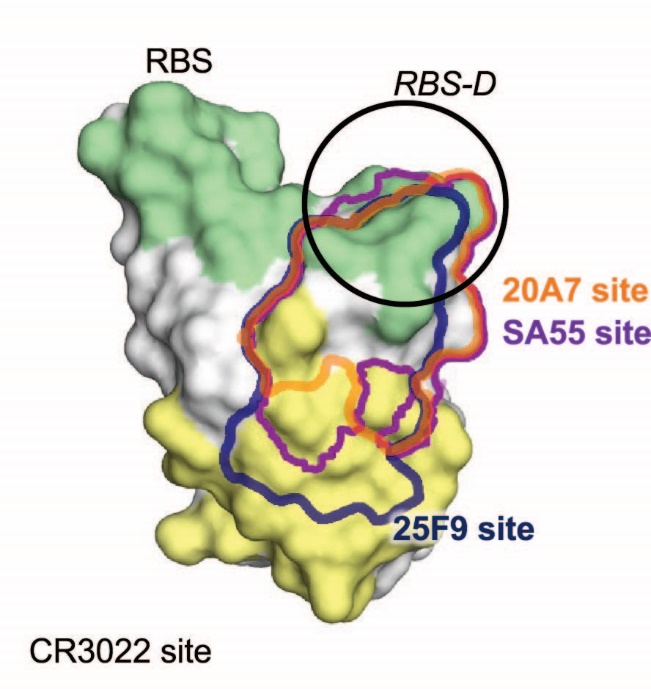


**Figure S9. Epitope map of SARS-CoV-2 RBD.**

The receptor binding site (RBS) and CR3022 epitope are shown in green and yellow, respectively. RBS-D is located on one corner of the RBS and is indicated by a black circle. The epitopes of 20A7, 25F9, and SA55 are outlined in orange, blue, and purple, respectively. Epitope residues are defined as BSA > 0 Å^2^ and calculated from RBD structures with CR3022 (PDB 6W41), ACE2 (PDB 6M0J), and SA55 (PDB 7Y0W), as well as 20A7 and 25F9 from this study.

**Figure S10. Structures of antibodies targeting the RBS-D/CR3022 region.**

The RBD is shown in a white surface with the RBS in green. Heavy and light chains of antibodies are shown in orange and yellow cartoons, respectively. Structures 20A7 and 25F9 are from this study, whereas DH1047 (PDB 7LD1), S2X259 (7M7W), SA55 (7Y0W), K398.22 (7TP4), and ADG20 (7U2D) are from the PDB.


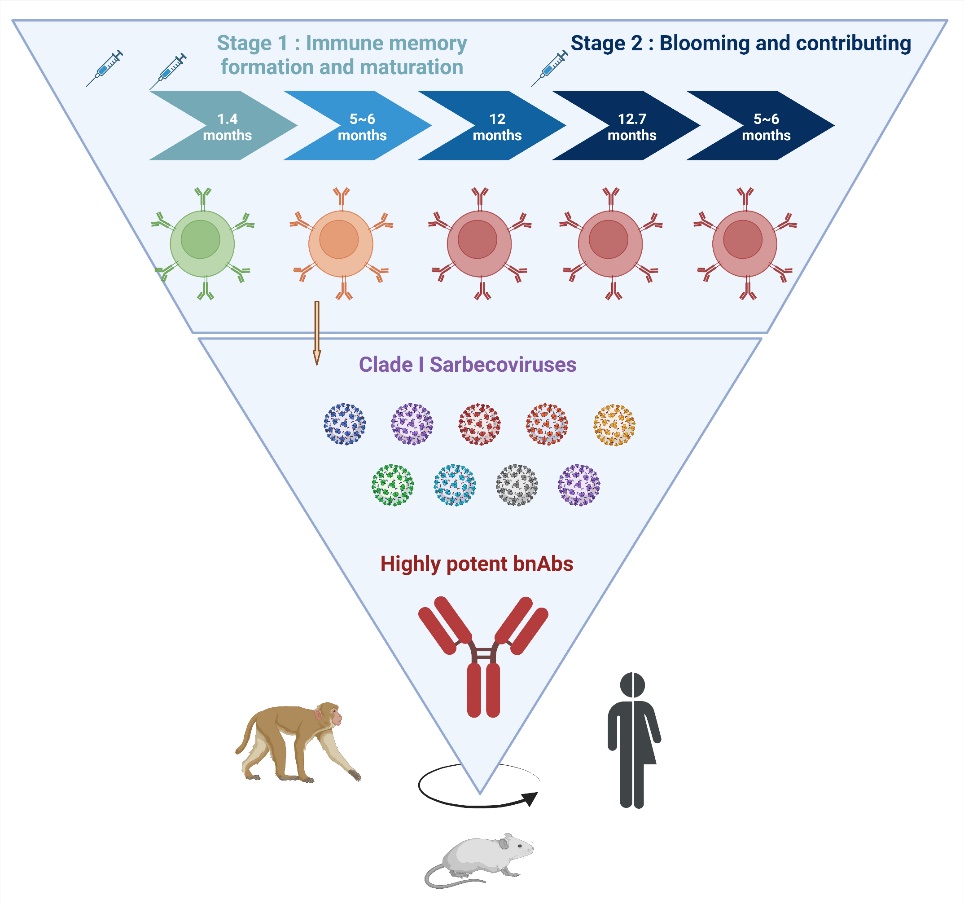


**Figure S11. A summary of current study.**

Memory B cells and antibodies from the memory B cell compartment evolve over time after vaccination. Memory B cells harboring potent and broadly neutralizing antibodies can be readily recalled after a booster vaccination or infection (“blooming”), which leads to the emerging pan-sarbecovirus bnAbs in serum (“contributing”).

**Supplementary Tables**

**Table S1. X-ray data collection and refinement statistics.**

| **Data collection** | 25F9 + wild-type  SARS-CoV-2 RBD | 21B6 + wild-type  SARS-CoV-2 RBD | 20A7 + wild-type  SARS-CoV-2 RBD | 20A7 + BA.2  SARS-CoV-2 RBD |
| --- | --- | --- | --- | --- |
| Beamline | APS23ID-D | APS23ID-B | APS23ID-B | SSRL12-1 |
| Wavelength (Å) | 1.0332 | 1.0332 | 1.0332 | 0.9795 |
| Space group | P 1 | P 2_1_ 2_1_ 2_1_ | P 2_1_ 2_1_ 2_1_ | P 2_1_ 2_1_ 2_1_ |
| Unit cell parameters |  |  |  |  |
| a, b, c (Å) | 92.5, 105.8, 118.2 | 72.7, 78.8, 131.8 | 44.8, 131.4, 174.1 | 44.5, 132.2, 170.5 |
| α, β, γ (°) | 83.1, 67.5, 64.0 | 90, 90, 90 | 90, 90, 90 | 90, 90, 90 |
| Resolution (Å) ^a^ | 50.0-3.05 (3.10-3.05) | 50.0-1.75 (1.78-1.75) | 50.0-2.58 (2.62-2.58) | 50.0-2.30 (2.34-2.30) |
| Unique reflections ^a^ | 69,365 (3,475) | 75,221 (7,384) | 33,431 (3,097) | 45,705 (4,413) |
| Redundancy ^a^ | 2.7 (2.6) | 11.7 (9.2) | 11.6 (7.0) | 11.8 (7.7) |
| Completeness (%) ^a^ | 98.6 (97.5) | 97.7 (96.7) | 99.2 (92.0) | 99.7 (97.3) |
| <I/σ_I_> ^a^ | 1.8 (0.4) | 20.9 (1.1) | 13.4 (1.0) | 20.9 (1.2) |
| *R*_sym_^b^ (%) ^a^ | 43.5 (>100) | 12.8 (93.5) | 17.0 (82.6) | 10.9 (>100) |
| *R*_pim_^b^ (%) ^a^ | 30.9 (>100) | 3.9 (30.3) | 5.1 (31.1) | 4.3 (42.4) |
| CC_1/2_^c^ (%) ^a^ | 87.7 (37.9) | 99.8 (80.5) | 99.1 (68.2) | 99.6 (75.6) |
| **Refinement statistics** | | |  |  |
| Resolution (Å) | 46.2-3.35 | 39.4-1.75 | 41.3-2.58 | 43.1-2.30 |
| Reflections (work) | 52,773 | 75,149 | 33,383 | 45,687 |
| Reflections (test) | 2,519 | 1,993 | 1,564 | 2,314 |
| *R*_cryst_^d^ / *R*_free_^e^ (%) | 27.2/31.9 | 19.4/22.1 | 22.4/24.8 | 19.6/22.6 |
| No. of antibody/RBD copies in ASU | 4 | 1 | 1 | 1 |
| No. of atoms | 19,708 | 5,459 | 4,911 | 5044 |
| RBD | 6,337 | 1,557 | 1,503 | 1,569 |
| Fab | 13,236 | 3,309 | 3,233 | 3,277 |
| Ligands ^f^ | 74 | 10 | 0 | 25 |
| Solvent | 62 | 583 | 175 | 194 |
| Average *B-*values (Å^2^) | 73 | 31 | 55 | 56 |
| RBD | 76 | 32 | 71 | 78 |
| Fab | 72 | 29 | 48 | 46 |
| Ligands ^f^ | 70 | 41 | N/A | 56 |
| Solvent | 36 | 40 | 50 | 52 |
| Wilson *B*-value (Å^2^) | 39 | 24 | 51 | 45 |
| **RMSD from ideal geometry** | | |  |  |
| Bond length (Å) | 0.012 | 0.007 | 0.002 | 0.002 |
| Bond angle (^o^) | 0.64 | 1.1 | 0.54 | 0.55 |
| **Ramachandran statistics (%) ^g^** | | |  |  |
| Favored | 95.6 | 96.2 | 96.2 | 97.6 |
| Outliers | 0.20 | 0.32 | 0.17 | 0.00 |
| **PDB IDs** |  |  |  |  |
|  | 8GB5 | 8GB6 | 8GB7 | 8GB8 |

^a^ Numbers in parentheses refer to the highest resolution shell.

^b^ *R*_sym_ = Σ*_hkl_* Σ*_i_* | I*_hkl,I_* - <I*_hkl_*> | / Σ*_hkl_* Σ*_i_* I*_hkl,i_* and R*_pim_* = Σ*_hkl_* (1/(n-1))^1/2^ Σ*_i_* | I*_hkl,i_* - <I*_hkl_*> | / Σ*_hkl_* Σ*_i_* I*_hkl,i_*, where I*_hkl,i_* is the scaled intensity of the i^th^ measurement of reflection h, k, l, <I*_hkl_*> is the average intensity for that reflection, and *n* is the redundancy.

^c^ CC_1/2_ = Pearson correlation coefficient between two random half datasets.

*^d^ R*_cryst_ = Σ*_hkl_* | *F*_o–_- *F*_c_ | / Σ*_hkl_* | *F*_o_ | x 100, where *F*_o_ and *F*_c_ are the observed and calculated structure factors, respectively.

^e^ *R*_free_ was calculated as for *R*_cryst_, but on a test set comprising 2.5% or 5% of the data excluded from refinement.

^f^ Bound ligands are SO_4_, ethylene glycol, glycerol, bicine, and PEG.

^g^ From MolProbity (*73*).

**Table S2. Primer list**

| Primer | 5’ – 3’ Sequence | Source |
| --- | --- | --- |
| 5′VH1.L1 | ATGGACTKGACCTGGAGG | Sundling *et al.,* 2012 (*60*) |
| 5'VH1/7 | GGACCTGACCCGGAGGATC | modified Wiehe *et al.,* 2014 (*61* |
| 5′VH2.L1 | ATGGACACGCTTTGCTCC | Sundling *et al.,* 2012 |
| 5′VH3A.L1 | ATGGAGTTKGGGCTGAGCTG | Sundling *et al.,* 2012 |
| 5'VH3_EXT2 | GGGGCTGAGYTGGGTTTTC | modified Wiehe *et al.,* 2014 |
| 5'VH3_EXT4 | TGGGCTGAGCTKGGTTTTY | modified Wiehe *et al.,* 2014 |
| 5′VH3B.L1 | ATGGAGTTTGKRCTGAGCTGG | Sundling *et al.,* 2012 |
| 5′VH3C.L1 | ATGGAGTCRTGGCTGAGCTG | modified Sundling *et al.,* 2012 |
| 5′VH3D.L1 | ATGGAGTTTGTGCTGAGTTTGG | Sundling *et al.,* 2012 |
| 5'VH4_EXT1 | ATGAAGCACCTGTGGTTCTBC | modified Wiehe *et al.,* 2014 |
| 5'VH4_EXT2 | ATGAAGCACCTGKGGTTCTTY | modified Wiehe *et al.,* 2014 |
| 5'VH5_EXT | ATGGGGTCAACTGCCMTCC | modified Wiehe *et al.,* 2014 |
| 5'VH6_EXT | ATGTCTGTCTCCTTCCTCATCGTC | modified Wiehe *et al.,* 2014 |
|  |  |  |
| 3' IgG_ext | TGTGCACGCCGCTGGTCAG | modified Wiehe *et al.,* 2014 |
|  |  |  |
| 5' VK1A_ext | TGTGACATCCAGATGACCCAG | New design |
| 5' VK1B_ext | GTGCCAGATGTGACATTCAG | New design |
| 5' VK2A_ext | ATGAGGCTCCCTGCTCAGCTC | New design |
| 5' VK2B_ext | CTSCCTGCTCWGCTCCTG | New design |
| 5' VK2C_ext | GATCCASTGGGGATGTTGY | New design |
| 5' VK3A_ext | CAGCACAGCTTCTCTTCCTCCTG | New design |
| 5' VK3B_ext | AGCTCGGCTTCTCTGCCTTCT | New design |
| 5' VK3C_ext | AGCTCAGCTTCTCTTCCTCCTGC | New design |
| 5' VK4A_ext | ATGGTGTCACAGACCCAAGTCTT | New design |
| 5' VK4B_ext | ATGGTGCTACAGACCCAGGTCCT | New design |
| 5' VK5_ext | GGTTCASCTCCTCAGCTTCCTC | New design |
| 5' VK6_ext | TTCTGCTSCTCTGGGTTCCAG | New design |
| 5' VK7_ext | ATGGGGTCCTGGGCTCCTT | New design |
|  |  |  |
| 3' CK_ext | GTCCTGCTCTGTGACACTCTCCT | modified Sundling *et al.,* 2012 |
|  |  |  |
| 5' VL1A-ext | ATGGCCTGGTYYCCTCTC | Sundling *et al.,* 2012 |
| 5' VL1B_ext | GGTCCTGGGCCCAGTCTG | New design |
| 5' VL2/7/10_ext | ATGSYCTGGRCTCTGCTCCTC | New design |
| 5' VL3A_ext | ACAGGTTCYGTGGTTTCYTCTG | New design |
| 5' VL3B_ext | ATGGCCTGGATTCCTCTCCT | modified Sundling *et al.,* 2012 |
| 5' VL3C_ext | ATGGCCTGGACCCYTCTCCT | modified Wiehe *et al.,* 2014 |
| 5' VL4A_ext | ATGGCCTGGGTCTCCTTC | Sundling *et al.,* 2012 |
| 5' VL4B_ext | ATGGCCTGGACCCCACTC | Sundling *et al.,* 2012 |
| 5' VL5/11_ext | ATGGCCTGGACHCCTCTCCT | modified Sundling *et al.,* 2012 |
| 5' VL6_ext | ATGGCCTGGGCTCCACTCC | Sundling *et al.,* 2012 |
| 5' VL8_ext | ATGGCCTGGATGATGCTTCT | modified Sundling *et al.,* 2012 |
| 5' VL9_ext | ATGGCCTGGGCTCCTCTGCT | modified Sundling *et al.,* 2012 |
|  |  |  |
| 3' CL_ext | TGTTGTTGCTCTGTTTGGAGGG | Zhang *et al.,* 2019 (72) |
|  |  |  |
| 5'VH1a_int | CAG GTS CAG CTG GTG CAR TC | New design |
| 5'VH1b_int | GAG GTC CAG CTG GTG CAG TC | New design |
| 5'VH1c_int | CAG CTG GTG CAA TCC GGG | New design |
| 5'VH2_int | CAG GTS ACC TTG AAG GAG TC | New design |
| 5VH3a_int | GAS GTG CAG CTG GTR GAG TC | New design |
| 5'VH3b_int | GAG GTG CAG CTG GTG GMG TM | New design |
| 5'VH3c_int | GAR GTG CAG TTG GTG GAG TC | New design |
| 5'VH4a_int | CAG STG CAG CTG CAG GAG TC | New design |
| 5'VH4b_int | CAG GTG AAG CTG CAG CAG TG | New design |
| 5'VH5/7a_int | SAG GTG CAG CTG GTG CAG TC | New design |
| 5'VH6_int | CAG GTG CAG CTG CAG GAG TC | New design |
|  |  |  |
| 3' IgG_int | GAA GTA GTC CTT GAC CAG GCA | New design |
|  |  |  |
| 5' VK1a_int | GACATCCAGATGWCCCAGKCT | New design |
| 5' VK1b_int | GACATTCAGWTGWCCCAGTCTC | New design |
| 5' VK2a_int | GATATTGTGATGAYCCAGACTCC | New design |
| 5' VK2b_int | TGGGGATGTTGTGATGACTCAG | New design |
| 5' VK3a_int | CCTGCTGCTCTGGMTCCCA | New design |
| 5' VK3b_int | CCTGCTACTSTGGCTCCCAG | New design |
| 5' VK3c_int | CCTGCTACCTTGGCTCCCAG | New design |
| 5' VK4_int | GACATTGTGATGACCCAGTCTCC | Zhang *et al.,* 2019 |
| 5' VK5_int | TCTCTGATGCCAGGGCAGAAA | New design |
| 5' VK6_int | CTCTGGGTTCCAGYCTCCA | New design |
| 5' VK7_int | GACATTGTGCTGACCCAGTCTC | Zhang *et al.,* 2019 |
|  |  |  |
| 3' CK_int | ATTCAGCAGGCACACAACAGAG | Zhang *et al.,* 2019 |
|  |  |  |
| 5' VL1a/10_int | CAGGCAGGGCTGACTCAG | modified Zhang *et al.,* 2019 |
| 5' VL1b_int | CAGTCTGTGCTGACDCAGC | modified Zhang *et al.,* 2019 |
| 5' VL2_int | TCCTGGGCTCAGKCTGCC | New design |
| 5' VL3a_int | TCYTCTGRGCTGACTCAGGA | New design |
| 5' VL3b_int | TCCTMTGAKCTGACTCAGCCAC | New design |
| 5' VL4/5/9/11_int | CWGCCTGTGCTGACTCAGYC | New design |
| 5' VL6_int | GAGGTTGTGTTCACTCAGCCC | modified Zhang *et al.,* 2019 |
| 5' VL7_int | CAGGCTGTAGTGACTCAGGAGCC | modified Zhang *et al.,* 2019 |
| 5' VL8_int | GAGACTGTGGTGACCCAGGAGC | modified Zhang *et al.,* 2019 |
|  |  |  |
| 3' CL_int | CTCCCGGGTAGAAGTCACTGATC | New design |

**Table S3 PCR programs.**

| **First round PCR program** | | |
| --- | --- | --- |
| **Temperature** | **Duration** | **Number of Cycles** |
| 95 ℃ | 5 minutes |  |
| 94-67-72 ℃ | 30-45-60 seconds | 3 |
| 94-64-72 ℃ | 30-45-60 seconds | 3 |
| 94-61-72 ℃ | 30-45-60 seconds | 3 |
| 94-58-72 ℃ | 30-45-60 seconds | 3 |
| 94-55-72 ℃ | 30-45-60 seconds | 3 |
| 94-52-72 ℃ | 30-45-60 seconds | 25 |
| 72 ℃ | 7 minutes |  |
| 4 ℃ | ∞ |  |
| **Second round PCR program** | | |
| **Duration** | **Duration** | **Duration** |
| 95 ℃ | 5 minutes |  |
| 94-58-72 ℃ | 30-45-60 seconds | 3 |
| 94-55-72 ℃ | 30-45-60 seconds | 3 |
| 94-52-72 ℃ | 30-45-60 seconds | 40 |
| 72 ℃ | 7 minutes |  |
| 4 ℃ | ∞ |  |

Data file S1. Raw, individual-level data for experiments where n<20.
